# Establishing Norm of Connected Speech Measures for Descriptive Discourses in Cantonese‐Speaking Adults

**DOI:** 10.1111/1460-6984.70055

**Published:** 2025-05-19

**Authors:** Anthony Pak‐Hin Kong, Chester Yee‐Nok Cheung, Cherie Wan‐Yin Wong

**Affiliations:** ^1^ Academic Unit of Human Communication, Learning, and Development (HCLD) Faculty of Education The University of Hong Kong Hong Kong Hong Kong; ^2^ The Aphasia Research and Therapy (ART) Laboratory The University of Hong Kong Hong Kong Hong Kong

**Keywords:** Cantonese, connected speech measures, descriptive discourse, norm

## Abstract

**Background and Objectives:**

Normative reference of the connected speech measures (both micro‐structural and macro‐structural) for descriptive discourse is fundamental to systematic discourse analysis because it provides an anchor for comparison. This study aims to establish a comprehensive normative reference for connected speech measures in Cantonese by analysing a wide array of micro‐ and macro‐structural measures, investigating the impact of age and education on these measures, and examining potential performance differences across various genres of descriptive discourse tasks.

**Method:**

The sample included 149 healthy Cantonese‐speaking adults who were categorized into three age groups (young, middle‐aged, and old) and two education levels (high and low). Speech samples were collected, transcribed, and analysed based on five descriptive discourse tasks in the Cantonese AphasiaBank, including two single‐picture descriptions (Flood and Cat rescue), two sequential picture descriptions (Broken window and Refuse umbrella), and one procedural description (Egg and ham sandwich).

**Results:**

Normative reference of multiple connected speech measures (such as type, token, noun‐verb ratio, Information Content Units [ICU] and Main Concepts [MC]) and lists of standard scoring criteria for the ICU and MC for the five tasks were presented. For age effect, statistical analysis revealed that the old group demonstrated less informativeness and fluency than younger groups across genres. Specifically in procedural description, the middle‐aged group showed superior verbal productivity compared to the other groups and informativeness compared to the old group. For education effect, the high‐education group outperformed the low‐education group in verbal productivity and informativeness across genres. Age‐education interaction was found in syntactic complexity measures across genres.

**Conclusions:**

These findings contribute to building a comprehensive normative reference for the evaluation of connected speech, providing a complementary tool for systematic and objective assessment.

**WHAT THIS PAPER ADDS:**

*What is already known on this subject*

- In the clinical context of assessment, discourse analysis is a crucial method for assessing language ability in individuals with acquired language impairments such as aphasia. Normative reference of the above‐mentioned connected speech measures (both micro‐structural and macro‐structural) is fundamental to systematic discourse analysis because it provides an anchor for comparison. Apart from discourse tasks from traditional aphasia assessment batteries, a large set of US English norms have also been established for discourse tasks included in the aphasia language databases of AphasiaBank.

*What this study adds*

- This is one of the very few reports in Cantonese that established a comprehensive and large‐scale normative reference for connected speech measures based on spontaneous oral discourse production from a sizeable group of native Cantonese speakers. Normative reference of an extensive array of connected speech measures, which includes multiple micro‐structural (linguistically‐based) and macro‐structural (content‐based) measures is provided.

*What are the clinical implications of this study?*

- Establishment of this linguistically and culturally specific normative reference enables more systematic and objective assessment of connected speech production in diseased populations, such as aphasia. Uncovering the effects of age and educational is crucial for theoretical inference of the impact of ageing and education level on language proficiency, as neglecting these effects can lead to biased comparisons when applying the norm to make clinical inferences.

## 1 Introduction

Discourse is defined as any language that is “beyond the boundaries of isolated sentences” (Ulatowska and Olness 2004, 300). As discourse analysis goes beyond isolated linguistic elements such as semantics, phonology, morphology, and syntax, it provides rich and valuable insights into the integral communicative competence of individuals by examining their language use within context and focusing on the functional aspects of language. In the clinical context of assessment, discourse analysis is a crucial method for assessing language ability in individuals with acquired language impairments such as aphasia. Fortunately, discourse tasks are receiving increased attention in recent years. To date, most of the clinically‐oriented standardized aphasia assessment batteries contain at least one discourse task; examples include the Western Aphasia Battery (WAB; Kertesz 2006), Minnesota Test for Differential Diagnosis of Aphasia (MTDDA; Schuell 1965), and the Diagnostic Aphasia Examination (BDAE; Goodglass and Kaplan 1993). However, regular clinical practice primarily focuses on isolated linguistic tasks, which refer to structured activities that assess specific linguistic skills, such as picture naming, in a controlled setting. These tasks often involve decontextualized exercises designed to evaluate proficiency in particular language components, rather than the broader dynamics of language use in real‐life situations (Bryant et al. 2016). Isolated language tasks may have limited ability to reflect interaction between linguistic components as well as cognitive factors that are presented in natural language use; thus, these tasks may not be able to precisely evaluate the actual language performance of people with aphasia (PWA) (Mayer and Murray 2003; Ulatowska et al. 2003; Herbert et al. 2008). In fact, PWA tend to be overrated in standardized tasks (Bryant et al., 2016). Essentially, evidence has shown that discourse‐eliciting tasks have a higher predictive power to everyday communication difficulties experienced by PWA than single‐word production tasks such as naming tasks (Herbert et al. 2008).

One of the most common discourse‐eliciting procedures used in clinically oriented assessment is through description of picture stimuli or an event, also known as descriptive discourse. In particular, descriptive discourse offers a relatively standardized but naturalistic way to evaluate the communicative competence of the speaker, which is clinically feasible and cost‐effective. It is important to establish a clear definition of various descriptive discourse tasks (also known as genres), which generally refer to activities that involve describing or explaining certain aspects of a picture or a process. More specifically, these tasks typically include (a) single‐picture descriptions, where the participant describes a single image; (b) sequential picture descriptions, where the participant describes a series of images in order; and (c) procedural descriptions, where the participant explains how to perform a specific task or procedure. There are several commonly used picture description tasks in standardized clinical assessment batteries, for example, the Cookie Theft picture (Goodglass and Kaplan 1993; Goodglass et al. 2001) from BDAE and the WAB Picnic scene picture (Kertesz 1982) are two of the most widely used single‐picture stimuli in the field of speech and language pathology. Applications of these tasks were found in various populations, including aphasia (e.g., Menn et al. 1994; Nicolas and Brookshire 1993), Alzheimer's disease (e.g., Giles et al. 1996; Kavé and Levy 2003), mild cognitive impairment (e.g., Choi 2009), brain injuries (e.g., Williams et al. 2010), right hemisphere stroke (e.g., Berube et al. 2022), as well as unimpaired elderly (e.g., Cooper 1990; Hux et al. 2023). Apart from using single‐picture stimulus, others also used sequential picture stimuli (Capilouto and Wright 2009; Kong 2011; Potechin et al. 1987) or procedural description task (Pritchard et al. 2015) to elicit verbal output.

One major and critical drawback of the picture description test in these traditional assessment batteries is rating subjectivity. More specifically, these tools generally use scoring grids to evaluate a speaker's performance, that is, speech‐language pathologists subjectively rate the performance based on scale ranging from *severe to within‐normal‐range*. This approach not only is difficult to precisely capture the detailed linguistic characteristics of speakers but is also subjected to problems of inter‐rater reliability and test‐retest reliability (Kong 2022). Furthermore, subjective ratings are insensitive to mild language impairment and subtle improvement resulting from intervention (Taler and Phillips 2008).

In order to achieve objective and comprehensive analyses of the speaker's performance, systematic quantitative discourse analysis is necessary, and it is often supported by semi‐automatic speech analysis software such as Computerized Language ANalysis (CLAN; MacWhinney 2000). The systematic quantitative analysis of descriptive discourse often focuses on two main aspects: the linguistically based and content‐based measures of spoken output (Kong 2022). The former analyses focus on language characteristics across various micro‐structural linguistic levels such as lexical use, syntactic complexity, verbal productivity, and fluency. Microstructural measures are considered to be more objective, in‐depth, sensitive to differences between impaired and healthy speakers, and easy to be computed through software. On the other hand, content‐based analyses focus on the ability to communicate meanings, themes, topics, and subject matter presented in the context, which are also known as macro‐structural measures. They often involve measurement of information units or main concepts (MC; Kong 2022). These objective measures not only can provide valuable information for assessment but also can guide the planning of treatment components and help monitor progress (Boyle 2020; Bryant et al. 2016; Kong 2009).

Noteworthily, normative reference is fundamental to systematic discourse analysis because it provides an anchor for comparison. A robust normative reference should be comprehensive, representative, and language‐specific. This means it should encompass a wide range of connected speech measures, including both micro‐structural and macro‐structural variables, cover various genres, and consist of a reference group that accurately reflects the target population, sharing the same native language and similar characteristics such as age or educational background. However, it is observed that many existing norm‐establishing studies do not meet these standards of comprehensiveness and representativeness, particularly in languages other than English.

### 1.1 Comprehensiveness: Measures and Genres

We noted that many norm‐establishing studies did not have a comprehensive array of the connected speech measures and genres in their norm. For instance, the norm of the updated version of Cookie Theft from the BDAE has been established in English (Berube et al. 2019), but the study only measured the total number of syllables and content‐unit‐based measures. While the norm of the WAB Picnic scene picture has been established in French‐Canadian (Boucher et al. 2022), they focused only on microstructural measures and information‐unit‐based macro‐structural measures. On the other hand, Richardson and Dalton (2016) established the norm of three discourse tasks extracted from the AphasiaBank database (http://talkbank.org/AphasiaBank/) but only focused on main‐concept‐based measures. Capilouto et al. (2005) established the norm for two single picture descriptions and two sequential picture descriptions extracted from Nicholas and Brookshire (1993), but they focused only on macro‐structural measures, not micro‐structural measures.

Inclusion of a wide range of connected speech measures is important, but the inclusion of various discourse genres is also essential. To our knowledge, most of the norm‐establishing studies did not incorporate all three types of descriptive discourse (e.g., Berube et al. 2019; Boucher et al. 2022; Capilouto et al. 2005; Richardson and Dalton 2016). In fact, different task genres may have different unique advantages in eliciting connected speech samples, which can potentially reflect different aspects of the individual's ability in discourse production. For example, sequential picture descriptions are believed to elicit longer, more lexically diverse production than single‐picture descriptions, while procedural descriptions may not always rely on visual stimuli because individuals can mentally visualize a sequence of actions they are familiar with, which could be a significant advantage for specific populations such as people with visual impairment (Bryant et al. 2016; Kong 2022). Studies also found differences in discourse production performance across genres when comparing between different age groups (Kintz et al. 2016). For example, Fergadiotis et al. (2011) found that lexical diversity might be affected by discourse genres. In picture description tasks, the stimuli likely provided adequate support for the narrative structure, aiding younger individuals while limiting the production of older adults. Consequently, no significant difference was found between the two groups. In contrast, the non‐pictorial stimuli, such as procedural discourse, offered more flexibility in selecting lexical items, resulting in higher lexical diversity in older adults when compared to the younger group. On the other hand, studies found that coherence is closely linked to the type of discourse used (Capilouto et al. 2005; Duong and Ska 2001; Glosser and Deser 1992; North et al. 1986; Wright et al. 2005). Recounts and procedural discourse are particularly resilient against age‐related declines (Glosser and Deser 1992; North et al. 1986). According to North et al. (1986) and Wright et al. (2005), these types of discourse are generally familiar and highly structured, allowing participants to effectively compensate for any age‐related decreases in their organizational discourse skills. In contrast, picture description tasks may provide a more accurate assessment of actual discourse abilities (Wright et al. 2005). Glosser and Desser (1992) noted that picture description tasks require participants to retain new information and establish connections with it, which demands greater cognitive skills, such as executive function and working memory, than simply relating well‐known concepts. Consequently, older adults with reduced storage capacity struggle more with picture descriptions than with recounts or procedural tasks. As the content of the connected speech sample depends on the nature of the eliciting task, a comprehensive assessment should include a more diverse set of discourse genres (Boyle 2020).

### 1.2 Representativeness: Age and Education

Apart from comprehensiveness, many norm‐establishing studies did not account for variables that might affect representativeness, such as age and educational background. In fact, providing age‐group‐specific or education‐level‐specific norms is not a common practice. For example, the English Cookie Theft norm established by Berube et al. (2019) did not divide the participants into age groups. While the French‐Canadian Cookie Theft norms established by Boucher et al. (2022) did provide age‐group‐specific norms, but they did not provide education‐level‐specific norms. This omission may undermine the representativeness of the norms, as individual's overall language ability is known to be significantly influenced by age (Kintz et al. 2016; Mackenzie 2000; North et al. 1986) and education (Mackenzie 2000).

Age‐related effects in discourse performance had been substantially studied. In a comprehensive review, Kintz et al. (2016) proposed that older adults might have specific difficulties in organizing discourse at the macro‐linguistic level, maintaining coherence, and tend to make more grammatical errors compared to younger adults. Studies also found age‐related declines in informativeness in picture description narratives (Marini et al. 2005; Wright et al. 2014) as well as efficiency in conveying main information (Mackenzie 2000). These changes may link to age‐related declines in non‐linguistic cognitive systems, such as attention, episodic memory, and working memory (Kintsch and van Dijk 1978; Kintz et al. 2016). Particularly, attention plays a role in selecting relevant information and suppressing irrelevant details during discourse organization (Kintz et al. 2016). Episodic memory is associated with retrieving specific events from memory to construct knowledge relevant to the discourse (Small et al. 1999), while working memory supports the continuous storage of information and its integration with newly incoming data (Baddeley 1996).

Education‐related effects in discourse performance were also evidenced. Early studies on both picture description and narrative discourse revealed that individuals with higher levels of education outperformed those with lower education in terms of informativeness and effective use of story structure (Juncos‐Rabadán 1996; le Dorze and Bédard 1998; Mackenzie 2000). Recently, Malcorra et al. (2022) conducted a more comprehensive investigation into the relationship between education level and discourse performance and found that higher education explained a better performance in microstructural and macrostructural connected speech measures. While the underlying reasons for education‐related effects on discourse performance remain unclear, it is widely agreed that education is strongly linked to cognitive reserve, a crucial protective factor that helps delay the onset of cognitive decline (Cotrena et al. 2016; Malcorra et al. 2022).

Given that both age and education can significantly influence an individual's discourse production abilities, having a normative reference that reflects the nuanced differences in normal performance across various age and education subgroups offers a crucial advantage for accurately detecting and assessing discourse deficits.

### 1.3 Aims

A large‐scale, comprehensive, and representative normative reference for connected speech measures of spontaneous descriptive discourse production in Cantonese is currently lacking. Therefore, the first aim of this study is to establish normative reference data for native Cantonese‐speaking populations that incorporates a comprehensive array of connected speech measures and multiple genres and provides age‐ and education‐subgroup normative reference. Specifically, we aimed to analyse a wide range of micro‐structural measures, including measures related to verbal productivity, lexical diversity, syntactic complexity, and fluency, as well as various macro‐structural variables including information‐unit‐based measures and main‐concept‐based measures. To achieve this, we also aimed to establish standard information content units (ICUs) and main concepts (MCs) for all the selected discourse tasks. This will facilitate uniform scoring of macro‐structural variables in upcoming clinical applications and research studies.

The second aim of this study is to investigate whether one's performance on these connected speech measures is affected by age and education. If so, it will further justify the necessity of dividing age and education subgroups in normative references. Based on previous studies, we expected there would be age and education effects in both micro‐structural variables and macro‐structural variables.

As discussed, various discourse genres may differ in their ability to elicit speech, which can influence a wide range of connected speech measures. Therefore, the third objective of this study is to investigate potential interactions between genre, age, and education in relation to different connected speech measures. Thereby, this study would analyse two single‐picture description tasks (one line drawing and one real photo), two sequential picture description tasks (one with 4 pictures and one with 6 pictures), as well as one procedural description task. It was expected that different genres might have unique sensitivity in identifying age and education effects in certain connected speech measures.

## 2 Method

### 2.1 Participants

The speech samples analysed in the present study were extracted from the Cantonese AphasiaBank (Kong and Law 2019), which can be accessed through registration at www.speech.hku.hk/caphbank/search/. Speech samples from a total of 149 native Cantonese‐speaking neurotypical adults of both genders with different age and education levels were included in this analysis. All participants were recruited in Hong Kong and met the inclusion and exclusion criteria of being at least 18 years old and native speakers of Cantonese. Exclusion criteria included acquired or developmental language impairments, mental illness, neurological impairments, traumatic brain injury, and visual or auditive deficits.

Participants were divided into three age groups: young (aged 18 to 39, *N* = 47, 24 women, *M_age_* = 26.32 years, *M_edu_* = 14.40 years), middle‐aged (aged 40 to 59, *N* = 48, 24 women, *M_age_* = 49.85 years, *M_edu_* = 12.38 years), and old (60 or above, *N* = 54, 28 women, *M_age_* = 66.07 years, *M_edu_* = 8.46 years); as well as High (*N* = 71) and Low (*N* = 78) education levels. Table 1 shows the distribution of participants by age, gender, and education subgroups. The cut‐off standard for high‐education level was defined as higher than secondary school or at least 14 years of education for younger groups and higher than primary school or at least 7 years of education for the oldest group. According to Kong and Law (2019), this cut‐off standard was based on the high illiteracy rate of native Cantonese elderly speakers in Hong Kong.

**TABLE 1:** Distribution of participants by age, gender, and education subgroups.

	Male		Female		
Age group	Low education	High education	Low education	High education	Subtotal
Young	11	12	12	12	47
Middle‐aged	16	8	15	9	48
Old	11	15	13	15	54
**Subtotal**	38	35	40	36	**149**

### 2.2 Original Procedures of Data Collection

Written informed consent was obtained from all participants. All participants were invited to perform eight monologic discourse production tasks across five genres (personal narrative, story‐telling, procedural description, single‐picture description, and sequential description), during which the participant was seated alone with the examiner in a quiet room. The present study focuses solely on descriptive discourse tasks, thus, five out of eight tasks were included, they were (1) *Flood*, a single‐picture description task of a colour real‐photo depicting a scene of rescuing someone in a flood, (2) *Cat rescue*, a single‐picture description task of a black‐and‐white line‐drawing of a ‘cat rescue’ scene, (3) *Broken window*, a sequential picture description task of a 4‐picture black‐and‐white line‐drawings of a ‘broken window’ scene, (4) *Refuse umbrella*, a sequential picture description task of a 6‐picture black‐and‐white line‐drawings of a ‘refuse umbrella’ scene, and (5) *Egg ham sandwich*, a procedural description task of describing how to prepare an ‘egg and ham sandwich’. All tasks were elicited using pictorial materials and prompted by the examiner with standard instructions. In addition, the task performances were video‐ and audio taped for subsequent transcription and analysis. Full details of data collection for the Cantonese AphasiaBank protocol can be found in Kong and Law (2019). Speech samples from each participant were compiled into a unified document with an assigned code. All personal demographic information dates and times of the recording were separately stored in a distinct master file.

### 2.3 Transcriptions

Recordings were orthographically transcribed by two research assistants with linguistical training using the format of Codes for the Human Analysis of Transcripts (CHAT; Macwhinney 2000) through a computerized program named Child Language Analyses (CLAN; Macwhinney 2000). The interrater reliability was greater than 99% based on the computation of 10% randomly selected samples (Kong and Law 2019).

### 2.4 Connected Speech Measures

Various connected speech measures were extracted and computed based on the transcriptions. The present study focused on two broad aspects of connected speech, which were micro‐structural variables and macro‐structural variables. Micro‐structural variables provide insights into the linguistic‐based characteristics of the connected speech, including lexical use, syntactic complexity, verbal productivity, and fluency, while macro‐structural variables provide insights into the content‐based characteristics of the connected speech, including the speaker's ability to convey meaning, themes, topics, and subject matter within a given context.

#### 2.4.1 Micro‐Structural Variables

All micro‐structural variables were calculated through the functions in CLAN software. Initially, the utterances were segmented based on the ‘one verb or clause per line’ principle except when a verb subcategorizes for a clause (see Kong and Law 2019 for details). Then, each lexical entry was annotated for part of speech (POS). Cantonese Romanized phonetic transcriptions and English gloss were automatically generated in the software. After that, micro‐structural variables were automatically computed using the EVAL instruction in CLAN software. We extracted four main categories of micro‐structural variables including (1) *Productivity* (duration, total utterances, mean length utterance‐words (MLU‐w), types, tokens, tokens per minute), (2) *Syntactic complexity* (verbs per utterance, noun‐to‐verb ratio, open‐to‐closed‐class ratio), (3) *Lexical diversity* (type‐token ratio; TTR), and (4) *Dysfluency* (retracing, repetitions). Remarkably, TTR (Templin 1957) was chosen over VocD (McKee et al. 2000) to estimate lexical diversity. This decision was driven by the fact that a considerable proportion of language samples in this study contained fewer than 50 tokens (the minimal requirement for VocD calculation; Yang et al. 2022). VocD is designed to be more robust to sample size differences, but it still relies on a minimum of 50 tokens for reliable calculations. When the sample size is too small, the random sampling process used in VocD becomes unstable, and the resulting value may not accurately reflect lexical diversity. TTR, while sensitive to sample size, can still be calculated for smaller samples with fewer than 50 tokens. It is straightforward to compute and does not rely on iterative sampling or complex probabilistic modelling, making it a practical alternative for small datasets.

#### 2.4.2 Macro‐Structural Variables

All macro‐structural variables were initially scored manually by the second author, who was a linguistically trained speech‐language pathologist. Both the variables related to ICU and variables related to MC were chosen. In particular, ICUs referred to ‘a word or group of words that carry a single unit of meaning regardless of their grammatical position in a sentence’ (Graesser et al. 2004). ICU‐related variables reflect the ability of a speaker to produce meaningful and relevant ‘key words’ in one's speech. MCs, on the other hand, were defined as ‘statements that provide an outline of the gist or essential information portrayed in the stimulus pictures or an outline of the essential steps in the procedures… and should contain one and only one main verb’ (Nicholas and Brookshire 1995, 148), simply put, the ‘gist’ of the content. MC‐related variables reflect the ability of a speaker to convey the ‘key ideas’ in one's speech regardless of whether one has produced the exact ‘key words’ or particular structures.

As there were no predetermined ICUs and MCs established for the discourse tasks we used, standard ICUs and MCs were established based on the majority of the content in our samples. To define majoritarian ICUs and MCs, the steps from the approach of Jensen et al. (2006) were adapted. First, all the transcriptions were propositionalized. A proposition can be defined as a combination of one predicate plus one or more arguments. The predicate may take the form of a verb, adjective, adverb, or sentence connector, while each argument of proposition represents a specific case relationship to its predicate, such as the agent, experiencer, instrument, object, source, or goal (Jensen et al. 2006). Second, each discourse task was divided into different thematic scenarios. Third, a list of core propositions was generated for each scenario based on the criterion that a proposition had to be produced by at least 20% of the participants; such invariant information was deemed critical for the effective transfer of information. Fourth, a list of standard ICUs was extracted from the core propositions in each scenario based on five categories: subjects, objects, places, actions, and others. Finally, a list of standard MCs was extracted from each scenario based on the criterion that the MC had to be produced by at least 60% of the participants (Kong and Wong 2018).

After the standard ICUs and MCs were determined, all the speech samples were scored accordingly. The scoring of MCs followed the steps of Main Concept Analysis (MCA; Kong 2009). Specifically, each speech sample was evaluated on whether all standard MCs were conveyed accurately and completely, each MC was then scored as one of the following four categories: (1) Accurate and Complete (AC) – 3 points, (2) Accurate but Incomplete (AI) – 2 points, (3) Inaccurate (IN) – 1 point, and (4) Absent (AB)—no point.

To check inter‐rater reliability of ICU and MC scoring, 5% of the samples were randomly extracted and re‐scored by the third author. Intraclass correlation coefficient (ICC) was used to assess the consistency of scoring made by both scorers. ICC values range from 0 to 1, with higher values indicating greater reliability. Typically, values below 0.40 indicate poor reliability, 0.40–0.59 indicate fair reliability, 0.60–0.74 indicate good reliability, and values above 0.75 reflect excellent reliability (Bartko 1966).

After scoring, a series of ICU‐related variables and MC‐related variables were calculated, including total ICUs, ICUs per second, ICUs per word, ICUs per utterance, MC scores, and ACs per minute. These variables reflected not only the speakers’ ability to convey key information but also the efficiency of the production.

### 2.5 Data Analysis

The statistical analyses for this study were performed using SPSS v25.0. A significant level was set at 0.05. There was adjustment of *p* values for multiple comparisons using planned Bonferroni correction (Weisstein 2004), as participant scores in micro‐structural and macro‐structural analyses were considered interdependent, the adjusted significant level was 0.025. To detect extreme scores for each connected speech measure, participants’ *z*‐scores of each variable were obtained, and extreme scores were transformed through 90% winsorization (Dixon 1980) to avoid significant distortion to the mean and standard deviation due to extreme outliers. The assumption of normality was verified based on Kolmogorov–Smirnov test (Lilliefors 1967). For the second objective, a series of two‐way between‐subject ANOVAs were conducted for each connected speech measure in each discourse task separately to evaluate the main effects of age, education, and their interaction. For those variables which had significant group differences but violated homogeneity of variance, the results were further verified by the non‐parametric Welch's tests to reduce the chance of committing Type 1 error (Tomarken and Serlin 1986). Post‐hoc tests were conducted to further compare the group differences. For the third objective, a series of three‐way mixed ANOVAs (genre, age, education) were performed to examine genre–age and genre–education interaction effects, aiming to clarify the potential differences in sensitivity to age and education effects across different genres.

## 3 Results

### 3.1 Normative Data of Connected Speech Measures

Relative to the first purpose of establishing normative references for the micro‐ and macro‐structural connected speech measures in Cantonese descriptive discourse, Tables S1–S5, respectively, summarize the winsorized descriptive statistics of these measures across the five discourse tasks: *Flood*, *Cat rescue*, *Broken window*, *Refuse umbrella*, and *Egg ham sandwich*. In general, productivity measures such as duration, total utterances, types, tokens, and tokens per minute were significantly affected by outliers with extreme scores, which led to abnormal kurtosis and skewness values and non‐normally distributed data. After 90% winsorization had been performed, all kurtosis values were significantly improved. Still, a relatively high positive skewness value was retained in most of the productivity measures. Syntactic complexity measures, MLU‐w, TTR, and all macro‐structural variables were relatively less affected by extreme scores. Retracing and repetition were rarely found in most of the participants, which also led to a relatively high positive skewness value and non‐normally distributed data in both variables.

### 3.2 Standard ICUs, MCs, and Interrater Reliability

To ensure consistent scoring of the macro‐structural variables in future clinical and research applications, this study defined standard ICUs and MCs for the five selected discourse tasks. All standard ICUs and MCs (both Cantonese‐original and English‐translated versions) are summarized in Tables S6–S10 and S11–S15, respectively. The ICCs for ICU and MC scoring were as follows: *Flood* had values of 0.702 and 0.858, *Cat rescue* had 0.909 and 0.908, *Broken window* had 0.960 and 0.873, *Refuse umbrella* had 0.899 and 0.926, and *Egg ham sandwich* had 0.868 and 0.894. These ICC values demonstrate an excellent level of inter‐rater reliability for both ICU and MC scoring across all five discourse tasks, except for the ICU scoring of *Flood*, which showed a good level of reliability.

### 3.3 Effects of Age and Education

The second purpose of this research is to explore the potential influence of age and educational background on individual performance across various measures of connected speech. The following is a summary of the results from two‐way ANOVAs conducted on winsorized data, additional ANOVAs, and subsequent post‐hoc testing for each of the five discourse tasks under description.

#### 3.3.1 Real‐Photo Single‐Picture Description—Flood

Table 2 shows the results of *Flood*, including the means, standard deviations, age main effects, education main effects, age–education interaction effects, and post‐hoc test results of all connected speech variables of the six groups (young and low‐education, young and high‐education, middle‐aged and low‐education, middle‐aged and high‐education, old and low‐education, old and high‐education).

**TABLE 2:** Means, standard deviations, and two‐way between‐group ANOVAs of connected speech measures of the real‐photo single‐picture description—‘*Flood*’.

	18–39 y.o. group		40–59 y.o. group		> 60 y.o. group					
Variables	Low ed. (*N* = 23)	High ed. (*N* = 24)	Low ed. (*N* = 31)	High ed. (*N* = 17)	Low ed. (*N* = 24)	High ed. (*N* = 30)	Age main effect *F*(2, 143)	Ed. main effect *F*(1, 143)	Interaction *F*(2, 143)	Post‐hoc
Micro‐structural										
Duration (sec)	31.90 (10.49)	36.96 (16.62)	36.65 (16.08)	42.16 (22.96)	30.67 (16.19)	47.13 (17.11)	1.275	10.532^***^	1.904	H > L^***^
#Utterances	9.15 (3.07)	12.50 (6.85)	11.66 (6.40)	13.21 (7.18)	8.88 (5.33)	14.28 (7.54)	0.743	10.699^***^	1.149	H > L^***^
MLU‐w	7.43 (1.52)	7.84 (1.34)	7.63 (1.58)	7.49 (1.41)	7.10 (1.66)	7.39 (1.49)	0.955	0.540	0.423	
Types	45.22 (13.26)	54.60 (18.58)	49.40 (17.54)	55.06 (21.90)	38.29 (14.30)	55.02 (20.86)	1.190	12.479^***^	1.213	H > L^***^
Tokens	69.89 (29.64)	94.46 (48.41)	86.50 (46.57)	98.12 (56.70)	61.52 (33.54)	103.75 (53.21)	0.719	11.756^***^	1.383	H > L^***^
Tokens/min	129.02 (31.11)	151.51 (23.48)	139.42 (32.62)	142.98 (34.01)	122.38 (29.78)	131.12 (36.91)	3.259	4.773	1.096	
Verbs/utterance	1.60 (0.35)	1.42 (0.37)	1.51 (0.47)	1.58 (0.37)	1.53 (0.33)	1.37 (0.33)	0.881	1.949	1.575	
Noun‐verb ratio	0.82 (0.25)	0.83 (0.25)	0.77 (0.30)	0.63 (0.14)	0.78 (0.25)	0.79 (0.22)	2.703	0.939	1.365	
O‐C ratio	1.24 (0.24)	0.93 (0.22)	0.94 (0.26)	1.06 (0.27)	1.17 (0.27)	0.95 (0.23)	1.440	10.820^***^	9.317^***^	L > H^***^
TTR	0.67 (0.08)	0.62 (0.10)	0.62 (0.11)	0.61 (0.12)	0.65 (0.08)	0.59 (0.13)	1.080	5.195^*^	1.024	L > H^*^
Retracing	0.57 (0.66)	0.58 (0.83)	0.42 (0.76)	0.53 (0.72)	0.50 (0.72)	0.53 (0.78)	0.203	0.184	0.049	
Repetitions	0.91 (1.20)	0.96 (1.16)	0.87 (1.26)	1.18 (1.59)	1.42 (1.41)	2.03 (1.85)	4.577^*^	1.803	0.497	O > Y^*^, O > M^*^
Macro‐structural										
Total ICUs	10.04 (2.51)	10.83 (2.71)	9.97 (2.42)	10.71 (2.95)	8.50 (2.28)	9.37 (3.09)	5.012^*^	3.201	0.007	Y > O^*^
ICUs/sec	0.35 (0.11)	0.34 (0.13)	0.31 (0.13)	0.32 (0.17)	0.33 (0.17)	0.22 (0.12)	3.072	2.856	2.907	
ICUs/word	0.17 (0.07)	0.14 (0.06)	0.14 (0.06)	0.15 (0.84)	0.16 (0.08)	0.11 (0.07)	0.627	3.755	1.637	
ICUs/utterance	1.20 (0.36)	1.06 (0.49)	1.07 (0.52)	1.05 (0.55)	1.13 (0.52)	0.82 (0.45)	1.338	3.703	1.089	
MC scores	10.13 (1.98)	10.17 (2.35)	9.77 (1.84)	9.65 (1.69)	8.08 (2.60)	8.47 (1.93)	11.127^***^	0.078	0.192	Y > O^***^, M > O^**^
ACs/min	6.92 (2.54)	6.36 (2.57)	6.03 (2.13)	5.89 (3.00)	6.24 (3.46)	3.89 (1.95)	4.635^*^	5.515^*^	2.560	Y > O^**^, L > H^*^

Abbreviations: AC = accurate‐and‐complete main concept; ed. = educational level; ICU = information content unit; M = middle‐aged; MC = main concept; min = minute; O = old; MLU‐w = mean length utterance (words); O‐C ratio = open‐to‐closed class ratio; sec = second; TTR = type‐token ratio; Y = young; y.o. = year‐old. The values are listed in the order “mean (standard deviation)”.

* = *p* < 0.025;

** = *p* < 0.005;

*** = *p* < 0.001.

Two‐way between‐subject ANOVAs revealed significant age effects in repetition, total ICUs, MC scores, and ACs per minute. Post‐hoc analyses revealed that the old group had significantly higher repetitions than the other groups; the young group had significantly higher total ICUs, MC scores, and ACs per minute than old group; whereas the middle‐aged group had a significantly higher MC scores than the old group only.

Significant education effects were found in duration, total utterances, types, tokens, open‐to‐close‐class ratio, TTR, and ACs per minute. Post‐hoc analyses revealed that the high‐education group performed significantly better than those in the low‐education group across all productivity measures such as duration, total utterances, types, and tokens. Surprisingly, the low‐education group had a higher open‐to‐close‐class ratio, TTR, and ACs per minute than the high‐education group.

A significant age‐education interaction effect was only found on the open‐to‐closed‐class ratio [(*F*(2, 143) = 9.317, *p* < 0.001]. Participants with low‐education tended to have higher scores than the high‐education group in both young and old groups, but an inverse effect was found in middle‐aged group.

Non‐parametric Welch's tests were conducted to verify every significant result which had violated homogeneity of variance based on Levene's test of equality. Results indicated that all the significant results were retained.

#### 3.3.2 Line‐Drawing Single‐Picture Description—Cat Rescue

Table 3 summarizes the results of *Cat rescue*. No significant age effect was found in any variable. Yet, significant education effects were found in duration, total utterances, types, tokens, tokens per minute, TTR, total ICUs, ICUs per word, ICUs per utterance and MC scores. Post‐hoc analyses revealed that the high‐education group performed significantly better than the low‐education group in most of the productivity measures, including duration, total utterances, types, tokens, and tokens per minute; they also performed significantly better in content‐related measures like total ICUs and MC scores. However, inverse effects were observed in TTR, ICUs per word, and ICUs per utterance.

**TABLE 3:** Means, standard deviations, and two‐way between‐group ANOVAs of connected speech measures of the line‐drawing single‐picture description—‘*Cat rescue*’.

	18–39 y.o. group		40–59 y.o. group		> 60 y.o. group					
Variables	Low ed. (*N* = 23)	High ed. (*N* = 24)	Low ed. (*N* = 31)	High ed. (*N* = 17)	Low ed. (*N* = 24)	High ed. (*N* = 30)	Age main effect *F*(2, 143)	Ed. main effect *F*(2, 143)	Interaction *F*(2, 143)	Post‐hoc
Micro‐structural										
Duration (sec)	41.61 (12.31)	47.67 (16.43)	49.58 (21.84)	56.41 (27.18)	38.77 (20.42)	54.63 (22.87)	2.031	7.786^*^	0.888	H > L^*^
#Utterances	14.30 (5.55)	18.21 (6.63)	18.13 (8.36)	21.35 (9.92)	12.33 (6.81)	19.53 (8.99)	3.349	13.319^***^	0.921	H > L^***^
MLU‐w	7.46 (1.26)	7.69 (0.88)	7.34 (1.51)	7.11 (1.20)	7.72 (1.57)	7.43 (1.37)	1.048	0.190	0.548	
Types	57.46 (13.73)	67.67 (13.87)	62.55 (17.28)	70.18 (21.23)	49.75 (15.09)	68.02 (20.21)	2.308	17.741^***^	1.308	H > L^***^
Tokens	107.04 (39.52)	137.13 (42.79)	127.03 (52.02)	146.88 (65.47)	88.92 (40.28)	141.93 (59.78)	2.232	16.429^***^	1.387	H > L^***^
Tokens/min	152.01 (27.82)	175.58 (25.62)	163.79 (25.98)	162.32 (32.23)	144.16 (27.32)	158.19 (27.93)	3.330	6.828^**^	2.375	H > L^**^
Verbs/utterance	1.54 (0.30)	1.39 (0.30)	1.33 (0.33)	1.32 (0.26)	1.43 (0.32)	1.36 (0.28)	2.457	2.180	0.632	
Noun‐verb ratio	0.82 (0.17)	0.82 (0.21)	0.86 (0.17)	0.82 (0.16)	0.93 (0.19)	0.84 (0.19)	1.820	2.302	0.971	
O‐C ratio	1.29 (0.32)	1.01 (0.19)	0.96 (0.18)	1.11 (0.23)	1.14 (0.26)	1.06 (0.24)	2.810	3.306	9.239^***^	
TTR	0.56 (0.09)	0.51 (0.07)	0.52 (0.09)	0.51 (0.11)	0.59 (0.08)	0.51 (0.09)	1.839	9.331^**^	1.739	L > H^**^
Retracing	1.13 (1.14)	0.88 (0.99)	0.58 (0.85)	0.76 (0.90)	0.54 (0.88)	1.00 (0.91)	1.482	0.667	1.795	
Repetitions	0.61 (1.08)	1.04 (1.42)	1.15 (1.30)	1.12 (1.17)	1.21 (1.52)	1.03 (1.24)	0.845	0.125	0.724	
Macro‐structural										
Total ICUs	24.87 (6.34)	28.42 (6.40)	28.03 (5.83)	29.59 (5.73)	22.50 (4.94)	28.60 (5.81)	3.792	14.542^***^	1.838	H > L^***^
ICUs/sec	0.63 (0.17)	0.63 (0.16)	0.63 (0.17)	0.61 (0.20)	0.67 (0.23)	0.58 (0.20)	0.009	1.212	0.889	
ICUs/word	0.25 (0.07)	0.22 (0.06)	0.24 (0.07)	0.23 (0.08)	0.27 (0.08)	0.23 (0.08)	0.598	5.367^*^	0.808	L > H^*^
ICUs/utterance	1.89 (0.60)	1.68 (0.52)	1.76 (0.58)	1.62 (0.63)	2.13 (0.78)	1.72 (0.75)	1.550	5.392^*^	0.557	L > H^*^
MC scores	22.89 (3.99)	25.21 (4.36)	24.84 (3.81)	25.94 (3.42)	22.25 (3.75)	25.20 (3.69)	2.435	10.842^**^	0.701	H > L^**^
ACs/min	11.46 (2.91)	11.26 (2.90)	11.43 (3.74)	10.81 (4.13)	12.69 (4.40)	10.53 (4.12)	0.207	2.515	0.957	

Note: The values are listed in the order ‘mean (standard deviation)’.

Abbreviations: AC = accurate‐and‐complete main concept; ed. = educational level; ICU = information content unit; M = middle‐aged; MC = main concept; min = minute; O = old; MLU‐w = mean length utterance (words); O‐C ratio = open‐to‐closed class ratio; sec = second; TTR = type‐token ratio; Y = young; y.o. = year‐old.

* = *p* < 0.025;

** = *p* < 0.005;

*** = *p* < 0.001.

Again, an interaction effect was found in the open‐to‐closed‐class ratio [(*F*(2, 143) = 9.239, *p* < 0.001]. Same as *Flood*, participants with low education tended to have higher scores than the high‐education group in both young and old groups, but an inverse effect was found in the middle‐aged group.

Similarly, Welch's tests were conducted for all the above significant results which had violated the assumption of homogeneity of variance; results revealed that all significant results were retained.

#### 3.3.3 Sequential 4‐Picture Description—Broken Window

Table 4 summarizes the results of *Broken window*. Two‐way ANOVAs revealed significant age effects in total utterances, MLU‐w, tokens per minute, verbs per utterance, total ICUs, ICUs per second, ICUs per word, ICUs per utterance, MC scores, and ACs per minute. For micro‐structural variables, post‐hoc analyses revealed that the middle‐aged group had significantly higher total utterances and MLU‐w than the young group; they also had significantly higher tokens per minute than the old group. Contrarily, the young group had significantly higher verbs per minute than the middle‐aged group. For macro‐structural variables, the old group performed significantly poorer than the young group across all variables; when compared to the middle‐aged group, they also performed significantly poorer in total ICUs, ICUs per second, MC scores, and ACs per minute. Also, significant education effects were found in types, tokens per minute, total ICUs, ICUs per second, and MC scores, where the performance of the high‐education group was significantly better than the low‐education group across all the above variables.

**TABLE 4:** Means, standard deviations, and two‐way between‐group ANOVAs of connected speech measures of the sequential 4‐picture description—‘*Broken window*’.

	18–39 y.o. group		40–59 y.o. group		> 60 y.o. group					
Variables	Low ed. (*N* = 23)	High ed. (*N* = 24)	Low ed. (*N* = 31)	High ed. (*N* = 17)	Low ed. (*N* = 24)	High ed. (*N* = 30)	Age main effect *F*(2, 143)	Ed. main effect *F*(2, 143)	Interaction *F*(2, 143)	Post‐hoc
Micro‐structural										
Duration (sec)	30.00 (12.52)	30.48 (8.95)	35.55 (14.65)	33.74 (16.35)	32.96 (13.03)	37.33 (12.79)	2.028	0.213	0.696	
#Utterances	9.00 (3.29)	10.96 (3.39)	12.92 (6.58)	13.24 (5.91)	9.67 (4.16)	12.73 (6.21)	4.065^*^	4.188	0.843	M > Y^*^
MLU‐w	7.56 (1.68)	7.60 (1.16)	6.85 (1.33)	6.75 (0.98)	6.99 (1.33)	7.25 (1.09)	4.251^*^	0.095	0.253	M > Y^*^
Types	42.65 (15.09)	47.29 (10.23)	48.39 (16.58)	49.65 (16.77)	38.77 (11.15)	50.30 (16.42)	1.306	5.606^*^	1.565	H > L^*^
Tokens	72.93 (36.88)	83.33 (27.68)	86.95 (41.36)	90.35 (45.88)	65.71 (28.23)	89.72 (40.08)	1.278	4.121	0.979	
Tokens/min	137.98 (33.24)	162.11 (26.30)	149.15 (30.60)	160.24 (28.95)	124.93 (25.91)	143.74 (27.39)	6.808^**^	13.969^***^	0.585	M > O^*^, H > L^***^
Verbs/utterance	1.46 (0.34)	1.32 (0.25)	1.22 (0.28)	1.19 (0.24)	1.34 (0.35)	1.30 (0.27)	4.665^*^	1.868	0.457	Y > M^*^
Noun‐verb ratio	1.10 (0.26)	1.08 (0.24)	1.07 (0.30)	1.12 (0.23)	1.03 (0.32)	1.02 (0.28)	0.980	0.005	0.209	
O‐C ratio	1.21 (0.24)	0.99 (0.18)	0.98 (0.23)	1.02 (0.22)	1.02 (0.22)	1.01 (0.20)	2.538	3.060	4.731^*^	
TTR	0.63 (0.10)	0.59 (0.08)	0.59 (0.09)	0.59 (0.11)	0.62 (0.08)	0.60 (0.08)	0.522	1.393	0.439	
Retracing	0.43 (0.73)	0.71 (1.00)	0.84 (1.00)	0.18 (0.39)	0.67 (1.01)	0.70 (0.88)	0.492	0.637	3.386	
Repetitions	0.30 (0.63)	0.33 (0.56)	0.52 (0.72)	0.59 (0.80)	0.42 (0.72)	0.53 (0.78)	1.302	0.378	0.048	
Macro‐structural										
Total ICUs	16.93 (4.29)	19.73 (4.23)	17.56 (4.44)	19.24 (3.19)	12.58 (3.65)	17.28 (4.06)	12.501^***^	21.117^***^	1.817	Y > O^***^, M > O^***^, H > L^***^
ICUs/sec	0.61 (0.17)	0.67 (0.15)	0.55 (0.17)	0.65 (0.22)	0.44 (0.20)	0.50 (0.18)	11.778^***^	5.868^*^	0.099	Y > O^***^, M > O^*^, H > L^*^
ICUs/word	0.27 (0.09)	0.25 (0.06)	0.23 (0.08)	0.24 (0.09)	0.21 (0.08)	0.22 (0.08)	4.387^*^	0.009	0.736	Y > O^*^
ICUs/utterance	2.04 (0.54)	1.90 (0.47)	1.64 (0.69)	1.62 (0.54)	1.46 (0.49)	1.61 (0.68)	7.274^***^	0.001	0.727	Y > O^**^, Y > M^*^
MC scores	17.83 (3.81)	20.54 (2.98)	18.77 (3.29)	20.65 (3.18)	14.96 (3.98)	18.87 (3.54)	9.076^***^	23.631^***^	1.050	Y > O^*^, M > O^**^, H > L^***^
ACs/min	12.38 (4.15)	13.75 (3.45)	11.72 (4.17)	13.63 (4.95)	8.76 (5.03)	10.18 (3.33)	11.393^***^	5.080	0.059	Y > O^***^, M > O^**^

*Note*: The values are listed in the order ‘mean (standard deviation)’.

Abbreviations: AC = accurate‐and‐complete main concept; ed. = educational level; ICU = information content unit; M = middle‐aged; MC = main concept; min = minute; O = old; MLU‐w = mean length utterance (words); O‐C ratio = open‐to‐closed class ratio; sec = second; TTR = type‐token ratio; Y = young; y.o. = year‐old.

* = *p* < 0.025;

** = *p* < 0.005;

*** = *p* < 0.001.

Similarly, open‐to‐closed‐class ratio test result revealed a significant interaction effect [(*F*(2, 143) = 4.731, *p* = 0.010]. Participants with low education tended to have higher scores than the high‐education group in both young and old groups, but an inverse effect was found in the middle‐aged group. All significant results were retained under non‐parametric Welch's tests.

#### 3.3.4 Sequential 6‐Picture Description—Refuse Umbrella

Table 5 summarizes the results of *Refuse umbrella*. Two‐way ANOVAs revealed significant age effects in total utterances, MLU‐w types, tokens, tokens per minute, open‐to‐closed‐class ratio, total ICUs, and MC scores. Post‐hoc analyses revealed that the old group performed significantly poorer than the young group in token per minute, open‐to‐closed‐class ratio, total ICUs, and MC scores; they also performed significantly poorer than the middle‐aged group in types, total ICUs, and MC scores. The young group also had a significantly higher open‐to‐closed class ratio than the middle‐aged group. No significant differences were found in total utterances, MLU‐w, and tokens between age groups.

**TABLE 5:** Means, standard deviations, and two‐way between‐group ANOVAs of connected speech measures of the sequential 6‐picture description—‘*Refuse umbrella*’.

	18–39 y.o. group		40–59 y.o. group		> 60 y.o. group					
Variables	Low ed. (*N* = 23)	High ed. (*N* = 24)	Low ed. (*N* = 31)	High ed. (*N* = 17)	Low ed. (*N* = 24)	High ed. (*N* = 30)	Age main effect *F*(2, 143)	Ed. main effect *F*(2, 143)	Interaction *F*(2, 143)	Post‐hoc
Micro‐structural										
Duration (sec)	34.63 (10.34)	36.17 (6.60)	38.34 (10.61)	38.29 (13.29)	30.54 (7.99)	38.02 (11.39)	1.974	3.094	1.894	
#Utterances	14.43 (4.93)	17.96 (4.90)	18.04 (6.98)	19.65 (7.24)	12.46 (3.97)	17.93 (7.11)	4.586^*^	12.255^***^	1.245	H > L^***^
MLU‐w	6.51 (0.84)	6.31 (0.92)	6.00 (0.73)	5.78 (0.82)	6.14 (1.11)	5.90 (0.92)	4.215^*^	2.137	0.005	
Types	55.74 (15.50)	60.33 (10.80)	58.10 (14.70)	60.35 (16.71)	44.71 (8.01)	56.92 (14.92)	5.582^*^	7.700^*^	1.793	M > O^*^, H > L^*^
Tokens	95.48 (33.72)	111.50 (25.77)	106.29 (38.11)	110.29 (42.18)	75.63 (19.50)	102.13 (37.30)	4.500^*^	7.636^*^	1.351	H > L^*^
Tokens/min	160.26 (24.38)	184.73 (24.79)	166.86 (26.50)	171.75 (25.84)	152.83 (29.83)	161.82 (30.22)	4.373^*^	7.904^*^	1.675	Y > O^*^, H > L^*^
Verbs/utterance	1.44 (0.32)	1.22 (0.24)	1.32 (0.25)	1.18 (0.22)	1.39 (0.32)	1.21 (0.22)	1.116	16.196^***^	0.223	L > H^***^
Noun‐verb ratio	0.84 (0.18)	0.77 (0.16)	0.67 (0.20)	0.76 (0.20)	0.74 (0.17)	0.70 (0.21)	3.494	0.025	1.945	
O‐C ratio	1.84 (0.40)	1.37 (0.30)	1.19 (0.24)	1.40 (0.39)	1.45 (0.36)	1.35 (0.36)	9.841^***^	4.595	11.600^***^	Y > O^*^, Y > M^***^
TTR	0.60 (0.07)	0.55 (0.06)	0.57 (0.08)	0.57 (0.09)	0.61 (0.08)	0.58 (0.09)	1.523	4.081	1.294	
Retracing	0.61 (0.84)	0.33 (0.64)	0.39 (0.62)	0.41 (0.62)	0.25 (0.53)	0.33 (0.61)	0.987	0.268	1.076	
Repetitions	0.39 (0.72)	0.33 (0.56)	0.58 (0.85)	0.47 (0.72)	0.38 (0.71)	0.50 (0.73)	0.572	0.014	0.358	
Macro‐structural										
Total ICUs	26.98 (4.66)	28.10 (4.00)	26.11 (4.51)	27.62 (4.31)	21.88 (4.28)	24.62 (4.99)	13.333^***^	5.675^*^	0.446	Y > O^***^, M > O^**^, H > L^*^
ICUs/sec	0.81 (0.17)	0.80 (0.12)	0.72 (0.19)	0.77 (0.20)	0.75 (0.24)	0.69 (0.18)	2.730	0.039	0.972	
ICUs/word	0.30 (0.07)	0.26 (0.05)	0.27 (0.07)	0.27 (0.08)	0.30 (0.07)	0.27 (0.08)	0.396	3.022	1.342	
ICUs/utterance	1.99 (0.40)	1.65 (0.40)	1.61 (0.51)	1.53 (0.49)	1.82 (0.48)	1.58 (0.61)	2.985	6.799^*^	0.782	L > H^*^
MC scores	30.43 (3.01)	31.40 (2.69)	30.43 (2.89)	31.47 (1.81)	27.75 (3.80)	29.35 (3.32)	10.339^***^	5.573^*^	0.167	Y > O^***^, M > O^**^, H > L^*^
ACs/min	18.73 (5.25)	17.83 (3.67)	16.59 (4.59)	17.99 (5.92)	18.28 (6.47)	16.23 (4.74)	0.620	0.368	1.405	

*Note*: The values are listed in the order ‘mean (standard deviation)’.

Abbreviations: AC = accurate‐and‐complete main concept; ed. = educational level; ICU = information content unit; M = middle‐aged; MC = main concept; min = minute; O = old; MLU‐w = mean length utterance (words); O‐C ratio = open‐to‐closed class ratio; sec = second; TTR = type‐token ratio; Y = young; y.o. = year‐old.

* = *p* < 0.025;

** = *p* < 0.005;

*** = *p* < 0.001.

On the other hand, significant education effects were found in total utterances, types, tokens, tokens per minute, verbs per utterance, total ICUs, ICUs per utterance, and MC scores. Post‐hoc analyses revealed that the high‐education group performed significantly better than the low‐education group in most of the above variables except verbs per utterances and ICUs per utterance where inverse effects were found.

Open‐to‐closed‐class ratio test results revealed an interaction effect [(*F*(2, 143) = 11.600, *p* < 0.001]. Once again, participants with low education had significantly higher scores than the high‐education group in both young and old groups, but an inverse effect was found in the middle‐aged group.

Similarly, Welch's tests were conducted for all the above significant results which had violated the assumption of homogeneity of variance; result revealed that all significant results were retained except the age effect of total utterance [(*F*(2, 95.871) = 2.840, *p* = 0.063].

#### 3.3.5 Procedural Description—Egg Ham Sandwich

Table 6 summarizes the results of *Egg ham sandwich*. Two‐way ANOVAs revealed significant age effects in duration, total utterances, types, tokens, tokens per minute, TTR, total ICUs, and ACs per minute. Post‐hoc analyses revealed that the middle‐aged group performed significantly better than the old group in most of the above variables including total utterances, types, tokens, tokens per minute, and total ICUs; they also had significantly higher total utterances than the young group. Surprisingly, the old group had a significantly higher TTR than the middle‐aged group.

**TABLE 6:** Means, standard deviations, and two‐way between‐group ANOVAs of connected speech measures of the procedural description—‘*Egg ham sandwich*’.

	18–39 y.o. group		40–59 y.o. group		> 60 y.o. group					
Variables	Low ed. (*N* = 23)	High ed. (*N* = 24)	Low ed. (*N* = 31)	High ed. (*N* = 17)	Low ed. (*N* = 24)	High ed. (*N* = 30)	Age main effect *F*(2, 143)	Ed. main effect *F*(2, 143)	Interaction *F*(2, 143)	Post‐hoc
Micro‐structural										
Duration (sec)	25.00 (9.35)	36.54 (15.02)	35.24 (14.53)	41.62 (15.65)	25.17 (12.05)	36.45 (14.74)	4.720^*^	17.974^***^	0.512	H > L^***^
#Utterances	8.13 (2.87)	13.92 (6.45)	13.13 (5.63)	16.53 (6.18)	8.33 (4.57)	13.10 (6.02)	8.168^***^	26.185^***^	0.548	M > Y^*^, M > O^*^, H > L^***^
MLU‐w	6.37 (1.24)	7.15 (1.22)	6.68 (1.25)	6.72 (1.33)	6.39 (1.24)	6.57 (1.15)	0.738	2.614	1.143	
Types	33.30 (9.58)	52.17 (16.05)	46.19 (14.39)	57.79 (16.39)	33.79 (12.69)	45.83 (16.03)	9.138^***^	34.730^***^	0.945	M > O^**^, H > L^***^
Tokens	52.50 (21.31)	100.85 (45.37)	87.37 (40.12)	111.50 (45.06)	52.81 (28.50)	84.92 (39.26)	8.391^***^	30.900^***^	1.243	M > O^**^, H > L^***^
Token/min	126.84 (27.55)	163.06 (26.78)	147.62 (28.10)	153.81 (25.59)	125.16 (26.36)	137.36 (31.63)	6.289^**^	15.106^***^	3.740	M > O^*^, H > L^***^
Verbs/utterance	1.19 (0.25)	1.09 (0.23)	1.08 (0.28)	1.06 (0.20)	1.30 (0.35)	1.14 (0.28)	3.594	4.655	0.777	
Noun‐verb ratio	1.00 (0.24)	1.00 (0.29)	1.07 (0.28)	1.06 (0.19)	0.94 (0.30)	0.99 (0.28)	1.584	0.173	0.166	
O‐C ratio	1.62 (0.61)	0.95 (0.21)	1.17 (0.43)	1.13 (0.46)	1.45 (0.57)	1.19 (0.32)	1.919	18.830^***^	5.752^**^	L > H^***^
TTR	0.67 (0.12)	0.56 (0.11)	0.57 (0.11)	0.55 (0.09)	0.68 (0.10)	0.58 (0.11)	5.011^*^	16.853^***^	2.278	O > M^*^, L > H^***^
Retracing	0.43 (0.66)	0.42 (0.58)	0.39 (0.67)	0.53 (0.80)	0.54 (0.78)	0.43 (0.68)	0.100	0.002	0.400	
Repetitions	0.17 (0.39)	0.44 (0.77)	0.58 (0.84)	0.65 (0.84)	0.58 (0.78)	0.80 (0.88)	3.381	1.989	0.201	
Macro‐structural										
Total ICUs	13.96 (4.37)	18.50 (6.00)	17.42 (4.85)	20.09 (5.70)	11.33 (3.47)	17.83 (5.41)	8.359^***^	29.741^***^	1.764	M > O^**^, H > L^***^
ICUs/sec	0.59 (0.15)	0.53 (0.14)	0.55 (0.19)	0.49 (0.15)	0.51 (0.19)	0.53 (0.18)	0.957	0.972	0.946	
ICUs/word	0.28 (0.08)	0.20 (0.08)	0.23 (0.09)	0.19 (0.06)	0.25 (0.09)	0.24 (0.09)	2.589	8.286^*^	2.506	L > H^*^
ICUs/utterance	1.80 (0.56)	1.45 (0.50)	1.51 (0.56)	1.27 (0.34)	1.58 (0.64)	1.58 (0.56)	2.444	4.630	1.355	
MC scores	10.00 (2.39)	10.67 (1.74)	10.03 (2.24)	10.35 (2.12)	8.63 (2.39)	10.57 (1.57)	1.786	7.861^*^	2.090	H > L^*^
ACs/min	8.69 (2.96)	6.61 (2.57)	6.51 (2.77)	5.49 (2.07)	7.50 (3.05)	6.66 (2.85)	4.171^*^	8.083^*^	0.700	L > H^*^

*Note*: The values are listed in the order ‘mean (standard deviation)’.

Abbreviations: AC = accurate‐and‐complete main concept; ed. = educational level; ICU = information content unit; M = middle‐aged; MC = main concept; min = minute; O = old; MLU‐w = mean length utterance (words); O‐C ratio = open‐to‐closed class ratio; sec = second; TTR = type‐token ratio; Y = young; y.o. = year‐old.

* = *p* < 0.025;

** = *p* < 0.005;

*** = *p* < 0.001.

On the other hand, significant education effects were found in duration, total utterances, types, tokens, tokens per minute, open‐to‐closed‐class ratio, TTR, total ICUs, ICUs per word, MC scores, and AC per minute. Post‐hoc analyses revealed that the high‐education group performed significantly better than the low‐education group in most of the above variables except open‐to‐closed‐class ratio, TTR, ICUs per word, and ACs per minute where inverse effects were found.

Open‐to‐closed‐class ratio test results revealed an interaction effect [(*F*(2, 143) = 5.752, *p* = 0.004]. Same as other discourse tasks, participants with low education had significantly higher scores than the high‐education group in both young and old groups, but no difference was found in middle‐aged group.

Similarly, Welch's tests were conducted for all the above significant results which had violated the assumption of homogeneity of variance, result revealed that all significant results were retained.

#### 3.3.6 Age–Education Interaction Effect

Age–education interaction effect was only observed in the open‐to‐closed‐class ratio and was consistently found across all genres. Participants with lower levels of education scored higher than those with higher levels of education in both the young and old age groups. However, an inverse effect was observed in the middle‐aged group.

### 3.4 Genre Interaction Effects

**FIGURE 1:**
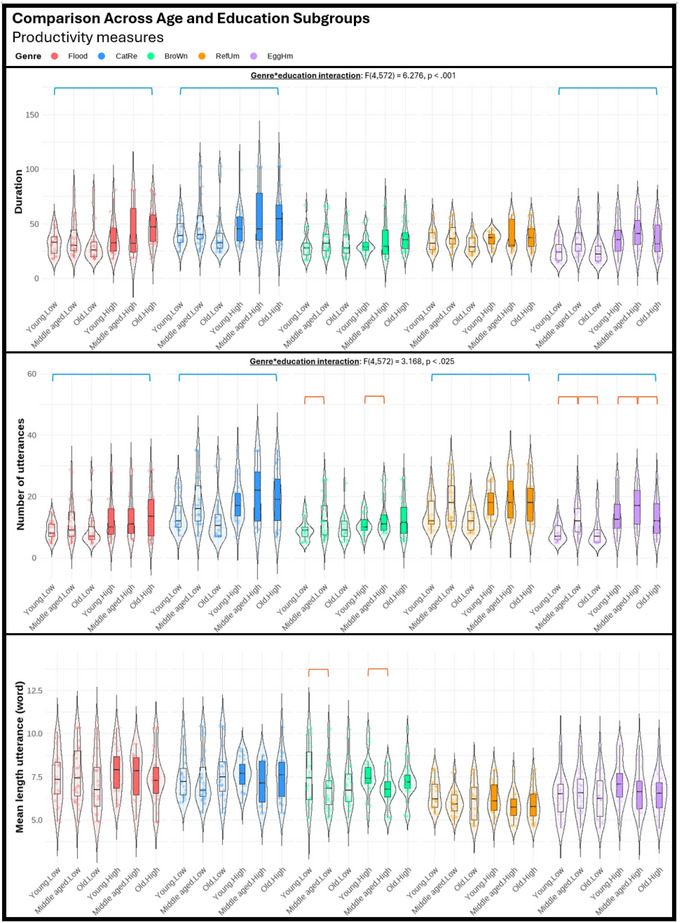
Violin plots showing productivity measures across age and education subgroups and genres. *Note*: Red and blue brackets indicate significant age and education effects respectively.

**FIGURE 2:**
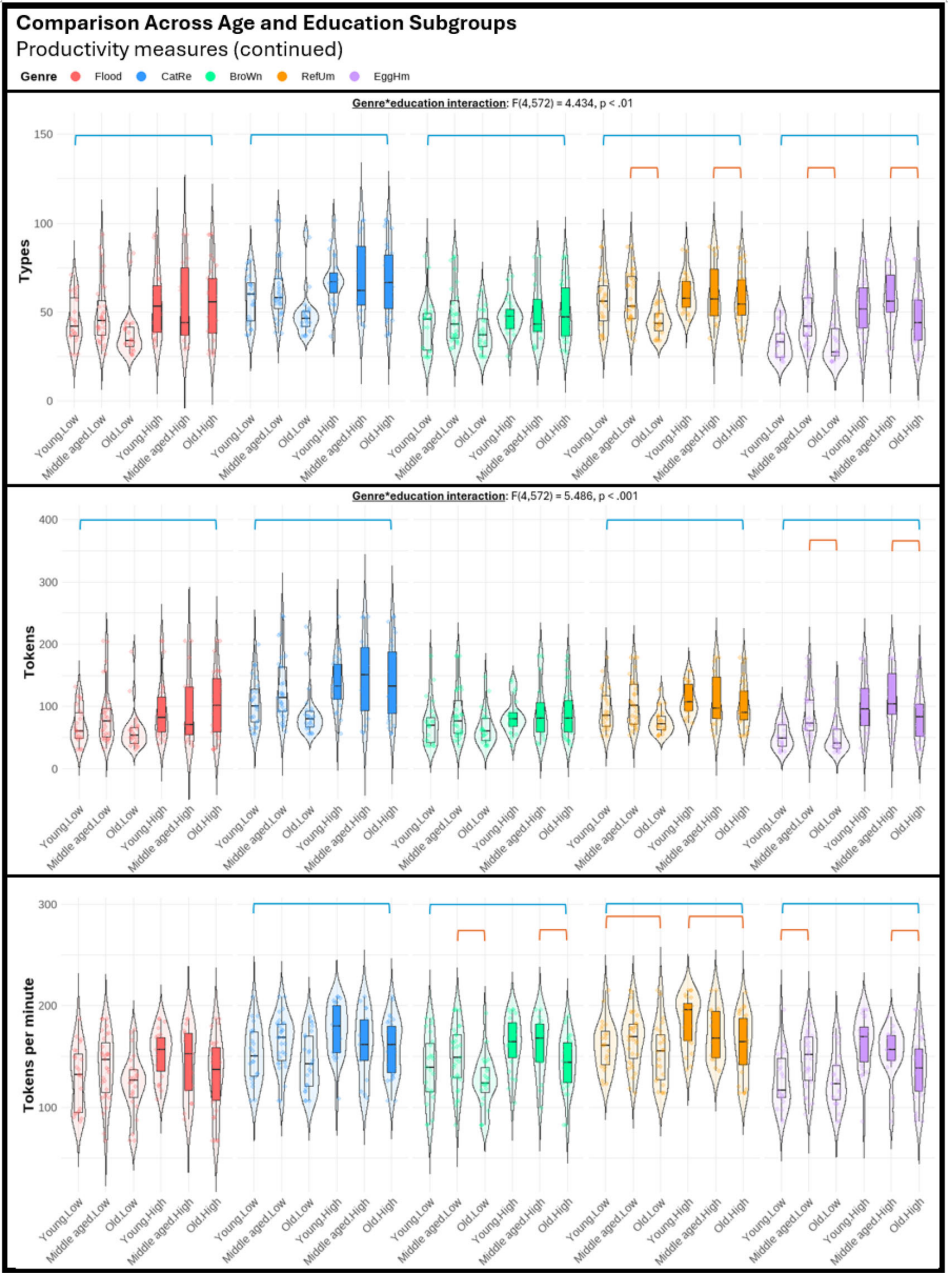
Violin plots showing productivity measures (cont.) across age and education subgroups and genres. *Note*: Red and blue brackets indicate significant age and education effects respectively.

**FIGURE 3:**
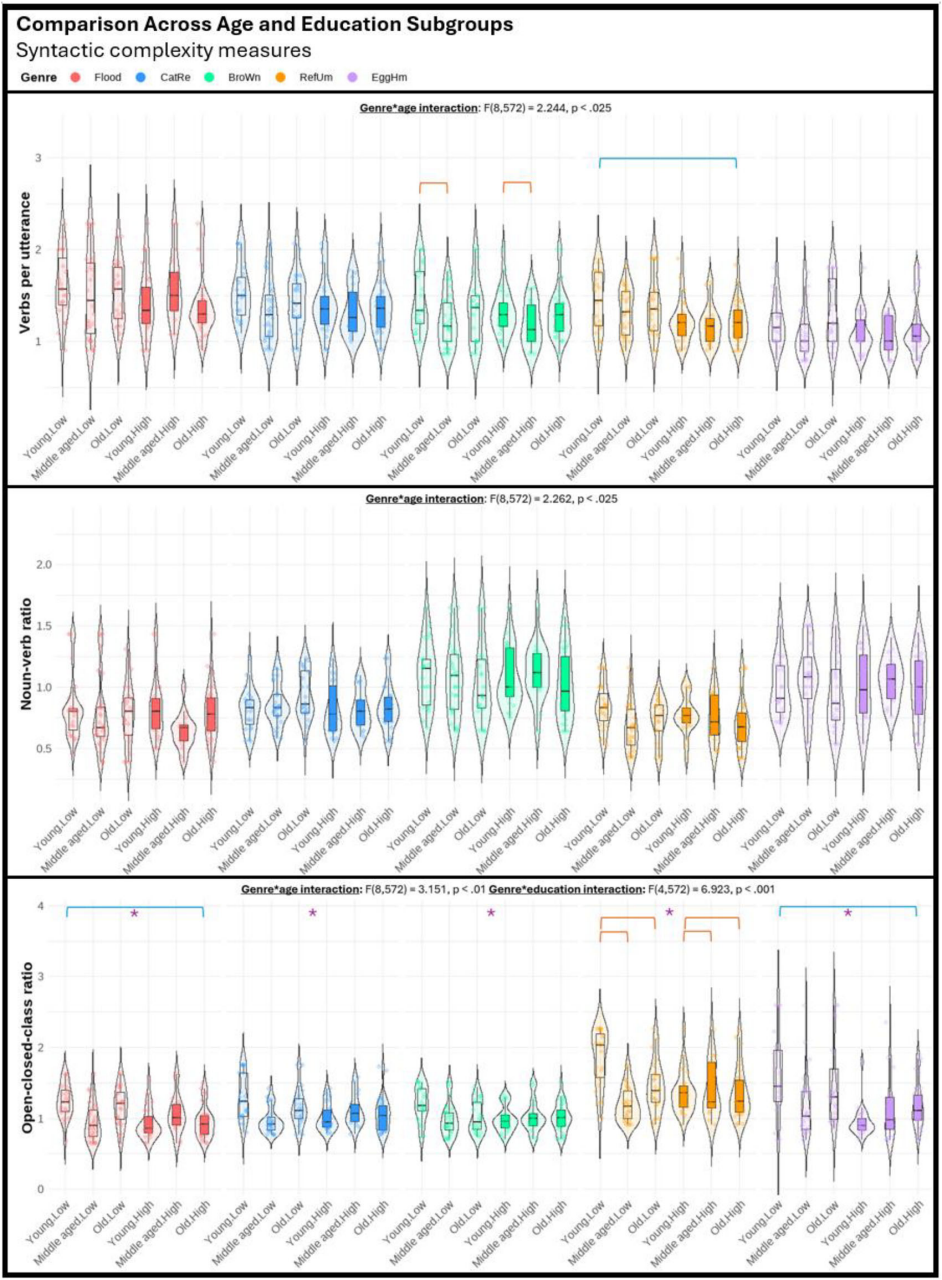
Violin plots showing syntactic complexity measures across age and education subgroups and genres. *Note*: Red and blue brackets indicate significant age and education effects respectively.

**FIGURE 4:**
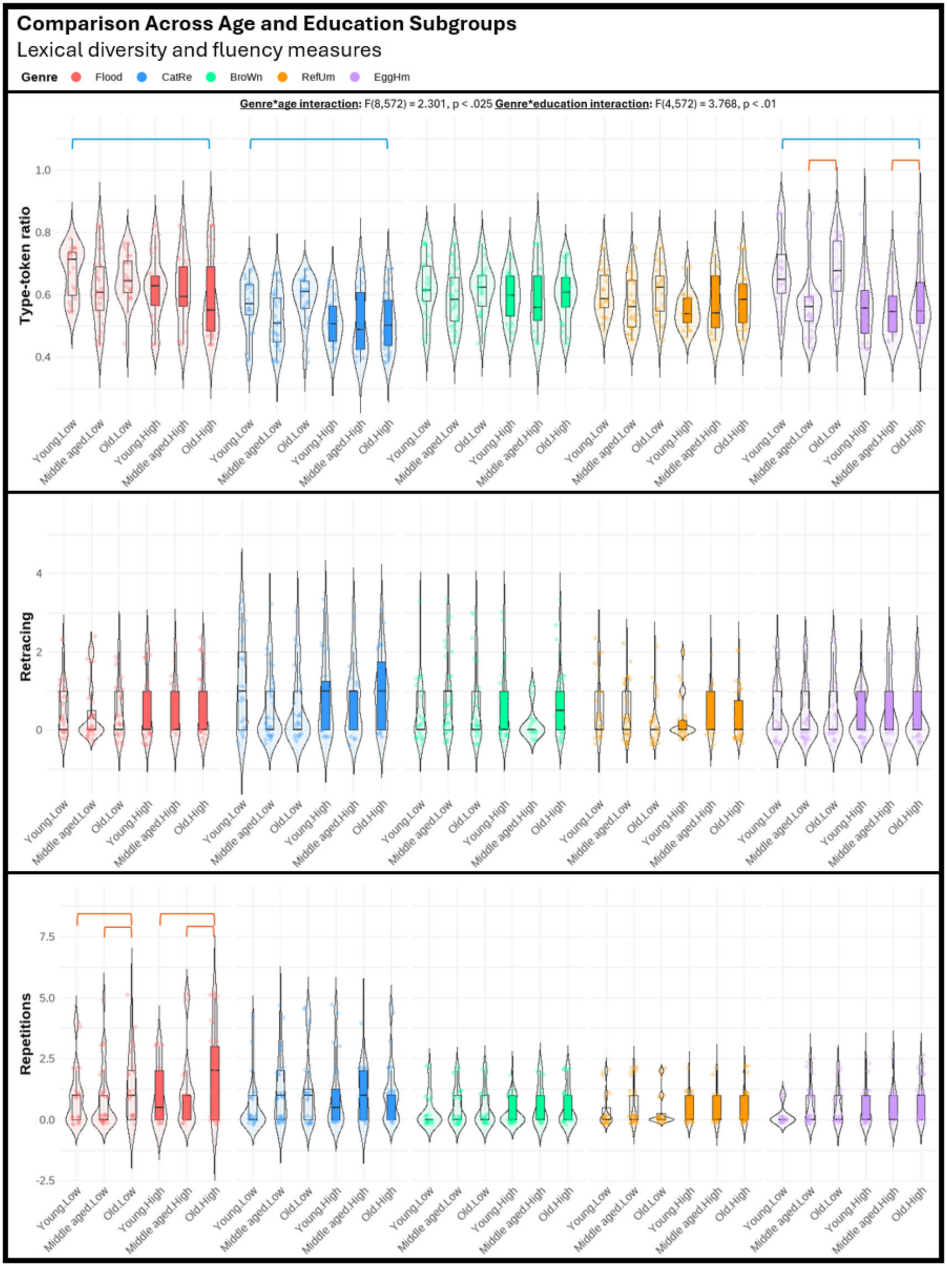
Violin plots showing lexical diversity and fluency measures across age and education subgroups and genres. *Note*: Red and blue brackets indicate significant age and education effects respectively.

**FIGURE 5:**
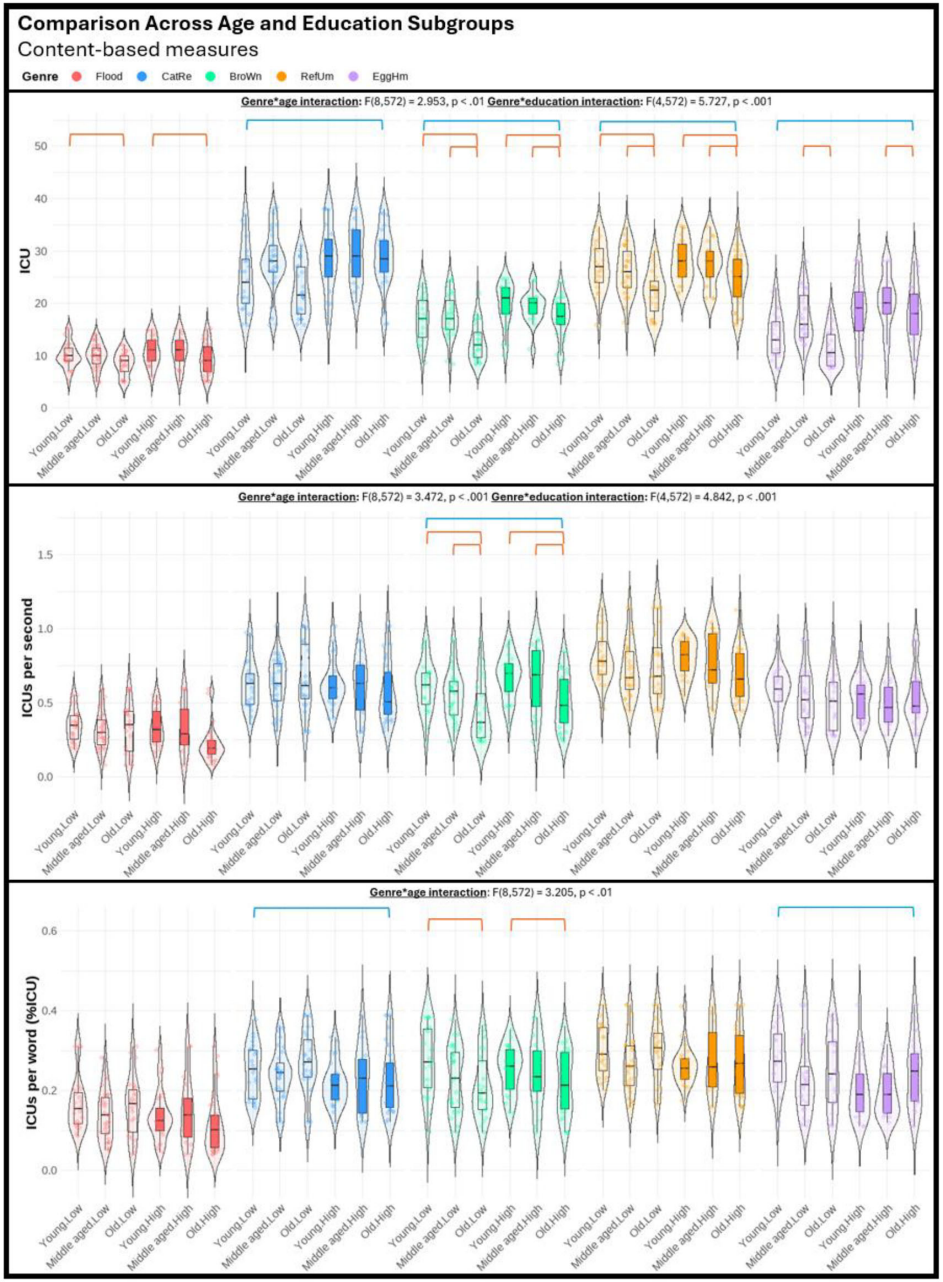
Violin plots showing content‐based measures across age and education subgroups and genres. *Note*: Red and blue brackets indicate significant age and education effects respectively.

**FIGURE 6:**
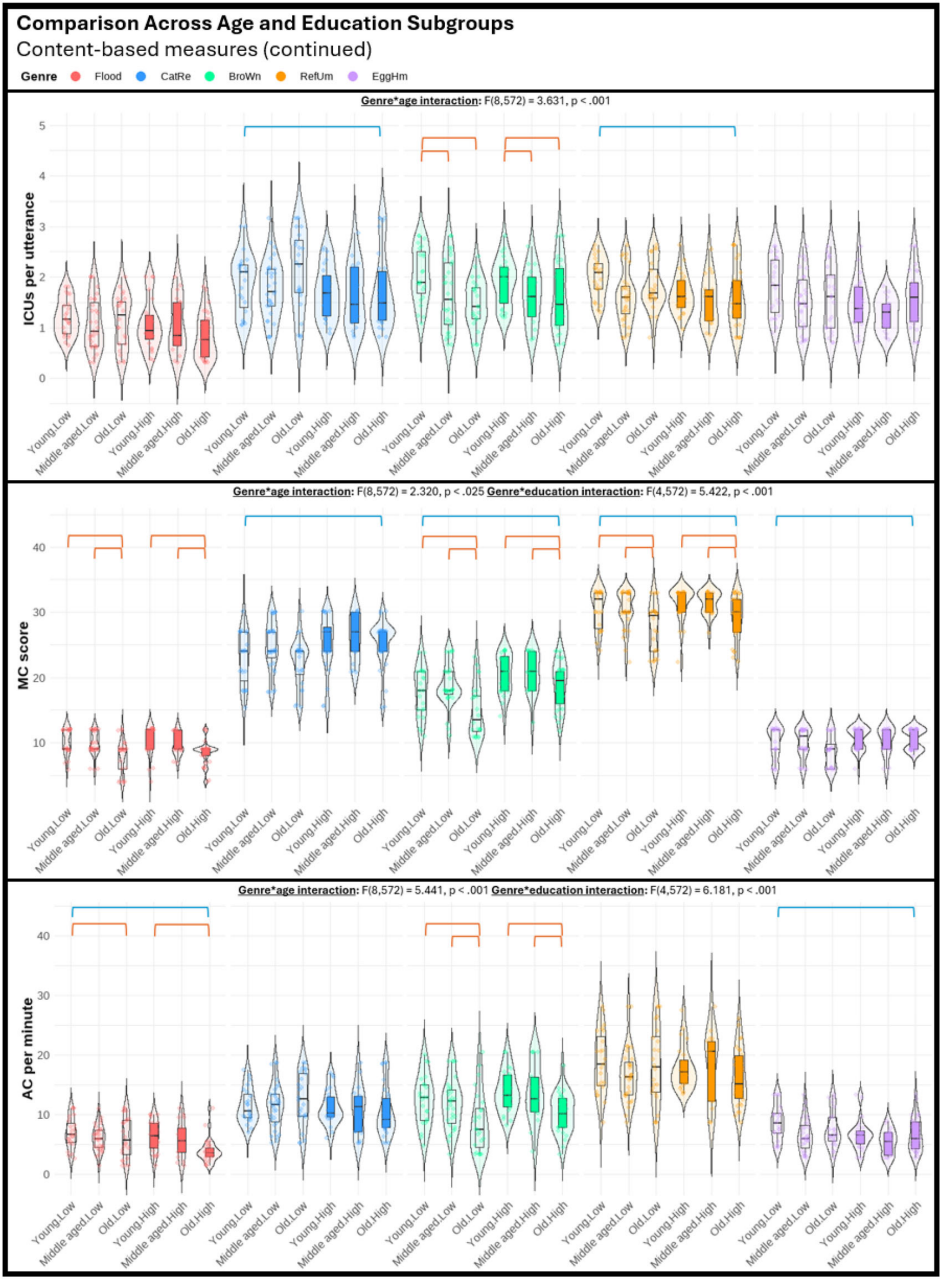
Violin plots showing content‐based measures (cont.) across age and education subgroups and genres. *Note*: Red and blue brackets indicate significant age and education effects respectively.

Regarding the third objective of the study, we performed a series of three‐way mixed ANOVAs (genre, age, education) to explore potential differences among various genres in their sensitivity to age and education effects. The violin plots displayed in Figures 1, 2, 3, 4, 5, 6 illustrate the comparisons among age and education subgroups across the different genres for each connected speech measure. A summary of all genre–age and genre–education interaction effects identified in the three‐way ANOVA is provided below.

#### 3.4.1 Genre‐Age Interactions

Significant genre–age interaction effects were found in syntactic complexity (verbs per utterance, noun‐verb ratio, open‐closed class ratio), lexical diversity (TTR), and all content‐based measures. No significant genre‐age interaction was found in any productivity measure, meaning that age effects observed in productivity measures did not vary across genres.

In micro‐structural measures, *Broken window* (sequential 4‐picture description) was more effective than the other genres in identifying age effects related to verbs per utterance. *Refuse umbrella* (sequential 6‐picture description) showed greater sensitivity in detecting age effects associated with the open‐closed‐class ratio, while *Egg ham sandwich* (procedural description) was more adept at identifying age effects in terms of TTR.

In macro‐structural measures, the old group consistently showed poorer ability to convey information in terms of ICU‐ and MC‐related measures, and such an effect was most noticeable in both sequential picture description tasks, but less pronounced in both single‐picture descriptions and procedural description (see Figures 5 and 6). Noteworthily, *Broken window* showed superior sensitivity in detecting ageing‐related decreases in total ICUs, MC score, and all efficiency‐related measures than other genres. Lastly, it is noteworthy that no significant age effect could be found in any connected speech measure in *Cat rescue*, the line‐drawing single‐picture description task.

#### 3.4.2 Genre‐Education Interactions

Significant genre–education interaction effects were found in productivity measures (duration, number of utterances, types, and tokens), syntactic complexity (open‐closed class ratio), lexical diversity (TTR), and content‐based measures (ICUs, ICU per second, MC score, and AC per minute).

In micro‐structural measures, higher productivity in the high‐education group was very consistent across genres, but *Broken window* was less sensitive in capturing differences in duration, number of utterances, and tokens. Higher open‐closed‐class ratios in the low‐education group were only detectable in *Flood* (real‐photo single‐picture description) and *Egg ham sandwich*. Higher TTR in the low‐education group was detectable in procedural description and both single‐picture descriptions, but not in both sequential‐picture descriptions.

In macro‐structural measures, the high‐education group consistently outperformed the low‐education group in absolute number of ICUs and MC score across genres except for *Flood*. This genre has significantly fewer ICUs and MCs overall, making it unable to distinguish between the abilities of the low‐education group and the high‐education group in conveying key information. Education effect in efficiency‐related measures was less consistent across genres (see Figures 5 and 6). In general, as the high‐education group tended to produce more utterances and words, a lower efficiency was reflected in ICUs per word and ICUs per utterance across multiple genres. However, *Broken window* was able to reflect better efficiency in the high‐education group in ICUs per second. This contrasting effect indicated that the high‐education group typically used longer language samples to convey information but did so in a shorter amount of time. Notably, these observations could not be identified within a single genre.

## 4 Discussion

This is one of the very few reports in Cantonese that established a comprehensive and large‐scale normative reference for connected speech measures based on spontaneous oral discourse production from a sizeable group of native Cantonese speakers. Establishment of this normative reference, being the primary purpose of this study, enables a more systematic and objective assessment of connected speech production in diseased populations such as aphasia. Comparing to other norm‐establishing studies, the norm provided in this study boasts several unique strengths. First, we present normative reference for a wide range of connected speech measures, encompassing various micro‐structural (linguistic‐based) and macro‐structural (content‐based) metrics. This approach enables a more thorough and detailed evaluation of connected speech metrics, overcoming the limitations found in many previous studies that focused on establishing norms with a more narrow scope (e.g., Berube et al. 2019; Boucher et al. 2022; Capilouto et al. 2005; Richardson and Dalton 2016). Second, this study examines the connected speech measures with different elicitation methods which enable discovery of genre‐specific characteristics which are impossible to be discovered in norm‐establishing studies concentrated on a single discourse task (e.g., Berube et al. 2019; Boucher et al. 2022; Hux and Frodsham 2023). Lastly, we systematically compare the performance of Cantonese speakers across different age groups and educational levels, enabling exploration of age, education, and interaction effects. To our knowledge, many norm‐establishing studies (e.g., Berube et al. 2019 and Boucher et al. 2022), did not categorize participants into age‐matched and education‐matched subgroups. Uncovering these effects is not only crucial for theoretical inference of the impact of ageing and education level on language proficiency, but also because neglecting age and education effects could lead to biased comparisons when applying the norm to make clinical inferences.

The norm established in this study emphasizes comprehensiveness and representativeness, which provides a complementary tool for clinicians and researchers to evaluate connected speech production in normal or diseased populations in a more objective way. Traditionally, due to time restrictions, the assessment of connected speech production primarily relies on qualitative rating scales (Bryant et al. 2016). However, with the increasing accessibility of semi‐automatic programs like CLAN, clinicians and researchers can now conveniently extract quantitative measures of connected speech production based on built‐in functions like EVAL instruction to examine variables reflecting verbal productivity (e.g., duration, total utterances, MLU, types, tokens, tokens per minute), syntactic complexity (e.g., verbs per utterance, noun‐to‐verb ratio, open‐to‐closed‐class ratio), lexical diversity (e.g., TTR), and dysfluency (e.g., retracing, repetitions). By comparing with the descriptive statistics of the norm, such as mean, standard deviation and interquartile range, examiners can gather valuable insights regarding the speaker's language proficiency. Moreover, as one of the advantages of the current norm, the examiner can also compare the performance of the speaker based on his/her age group and education level. Based on our findings, age and educational level significantly influence numerous connected speech measures, therefore, considerable discrepancies can be anticipated when an individual's performance is compared to the general norm and the norms of the subgroups. This is particularly useful when assessing young patients, as many of the established norms were based on older samples (e.g., Boucher et al. 2022).

Furthermore, this multiple‐genre norm can provide supplementary reference value for other norm‐referenced assessment tools that are limited to a single genre. For example, the Western Aphasia Battery was limited to single‐picture description (Kertesz 2006), while the MCA was limited to sequential picture description (Kong 2009). Fundamentally, different genres can elicit diverse responses that significantly impact the ensuing connected speech measures. For example, reports suggest that using sequential pictures is more conducive to prompting longer sentences, less ‘item listing’, and higher lexical diversity from individuals with aphasia than utilizing a single picture stimulus (Capilouto and Wright 2009; Potechin et al. 1987). On the other hand, procedural descriptions include a series of steps or actions to perform a task, which may potentially elicit more action verbs and connectives. It is possible that certain elicitation methods may authentically capture the true ability of individuals with certain characteristics. In essence, the introduction of this multi‐genre norm enriches our understanding and analysis of connected speech, allowing for a more comprehensive and precise assessment that addresses the limitations of single‐genre tools and captures the diverse responses elicited by different genres.

The second aim of this study is to investigate the impact of age and educational background on individual performance across various measures of connected speech. Our results show that healthy ageing has a significant negative impact on the accuracy and efficiency of conveying relevant information content as well as fluency during descriptive discourse production. These results are consistent with available literature in other languages (e.g., Boucher et al. 2022; Capilouto et al. 2016), which also demonstrated reduced communication efficiency and fluency in the older age group during descriptive discourse production. The reduction of connected speech performance observed in older adults can be attributed to various factors. Studies underscore that healthy ageing is related to declines in perception, processing speed, working memory, and controlled attention (Craik and Rose 2012). Within a cognitive processing framework, these changes impair both encoding and retrieval processes, leading to difficulties in carrying out self‐initiated mental operations (Craik and Rose 2012). According to the interactive‐construction model of discourse (Kintsch and Dijk 1978), discourse construction relies on four levels of representation: *surface* (combining linguistic units to form words and sentences), *semantic* (expressing concepts and the links between them), *situational* (depicting relationships between concepts in a described situation), and *structural* (organizing conceptual units). The cognitive decline associated with ageing can disrupt these levels of discourse production in multiple ways. For instance, working memory decline may impact the semantic level by hindering the ability to retain and connect micro‐propositions to form a cohesive gist (Kintz et al. 2016). Similarly, organizing a hierarchical structure of semantic elements often requires complex cognitive skills, such as executive functions and controlled attention, which can be reduced with age, thereby affecting the situational and structural levels of discourse. Consequently, these age‐related cognitive declines may manifest in connected speech as increased repetitions (as observed in our data and consistent with Boucher et al. 2022) and reduced efficiency in conveying key information (as supported by our findings, Boucher et al. 2022, and Capilouto et al. 2016).

Remarkably, a novel finding in our study is that the age‐related deficits in conveying key information were most prominently captured in sequential picture description tasks compared to other genres in this study. This phenomenon may potentially be related to increased cognitive loading (e.g., higher demand of executive function) and higher demand of stimulus understanding (including the motives of the characters and the narrative elements of the event) during sequential picture description task. Furthermore, previous studies have demonstrated older adults tended to express more irrelevant contents than younger adults (Arbuckle et al. 2000; Capilouto et al. 2016; Cooper 1990; Glosser and Deser 1992; James et al. 1998; Juncos‐Rabadan et al. 2005; Marini et al. 2005; Pushkar et al. 2000). In this sense, sequential picture description may be more sensitive to capturing this kind of coherence issue than other genres but warrants further investigation.

Another interesting finding of the present study is that the middle‐age group exhibited the highest levels verbal productivity and informativeness compared to the other cohorts, and such an effect was predominantly observable in the procedural description task. This aligns with findings from another similar study by Capilouto et al. (2016), which also made similar division of age groups and discovered an optimal language use among the middle‐aged group in both single‐picture and sequential‐picture description. This trend could potentially be attributed to the increase in experience and world knowledge associated with age (Hulit et al. 2011). While the advantage of accumulated crystallized intelligence tends to increase with age, the negative impact of typical cognitive decline associated with healthy ageing eventually supersedes this advantage. Consequently, language proficiency may peak during middle age, followed by a noticeable reverse in the oldest group. Hence, the age effects identified in this study suggest a rather complex, non‐linear relationship between age and language proficiency. Nonetheless, the reason behind the superior performance of the middle‐aged group specifically seen in the procedural description task certainly merits additional investigation.

On the other hand, we also found that an individual's educational level has a substantial positive impact on verbal productivity and informativeness, while such an effect was consistent across all genres. This finding aligns with previous studies which demonstrated a positive effect of individual's education level on communicative adequacy (e.g., Mulder and Hulstijn 2011). Unexpectedly, we noted a trend where the group with a lower education level performed better in areas like TTR, open‐to‐closed classes ratio, and some efficiency‐related macro‐structural variables such as ICUs per word, ICUs per utterance, and ACs per minute. One possible explanation of such result is that the high‐education group tended to have a longer speech sample, which leads to a larger denominator in measures like TTR and efficiency‐related macro‐structural variables. Furthermore, it is known that the overall length of a speech sample can significantly affect TTR (McCarthy and Jarvis 2010). As the length of a text increases, the probability of new words (types) appearing tends to decrease—most words in a large text have likely already been used earlier in the text. Therefore, as the text gets longer, the number of tokens increases faster than the number of types, leading to a lower TTR. This is often referred to as the ‘lexical diversity paradox,’ where longer texts, despite potentially having a more diverse vocabulary, may appear to have less lexical diversity when measured with TTR (Covington and McFall 2010). At first glance, it may seem counterintuitive that individuals with higher education appeared less efficient at conveying key information. However, our study on sequential picture description (specifically the *Broken window* task) revealed that the highly educated group actually took less time to communicate key information compared to the less educated group, despite using longer speech samples. This finding supports our argument that evaluating language ability requires a variety of connected speech measures across multiple genres. In summary, these findings underscore the complex interplay between education level and various aspects of discourse production, and the choice of connected speech measures may affect the interpretation of the final results. Future studies may use adjusted measures of lexical diversity and communication efficiency to uncover the above issue.

Moreover, the age‐education interaction effect was solely noticed in the open‐to‐closed‐class ratio which represents syntactic complexity. Result showed that this trend remained constant across all discourse tasks. Individuals with less education outperformed those with more education in both young and old groups, contrarily, the middle‐aged group displayed a reverse of this effect. This counterintuitive finding is not easy to explain. This unique interaction effect might be influenced by numerous factors, such as differences in cognitive abilities, communication habits, and the specific demands of different discourse tasks, among other potential factors. The precise reasons for this finding would require further investigation and analysis to fully understand.

Lastly, the third aim of this study is to examine how genre interacts with the age and education effects observed in our results. We discovered that different genres vary in their sensitivity to detecting these effects across various connected speech measures. Notably, sequential picture description demonstrated greater sensitivity in identifying age‐related declines in content‐based measures, while older adults showed relatively compatible performance in single‐picture description and procedural description tasks. This finding aligns with the results from North et al. (1986) and Wright et al. (2005) that discourse types relying less on pictorial stimuli tend to be more resistant to age‐related declines in language proficiency. For example, procedural discourses are typically familiar and highly structured, enabling participants to effectively compensate for age‐related reductions in their discourse organization skills. On the contrary, discourse tasks with higher demand on picture stimuli, such as sequential picture description, require participants to select useful information, retain the information, and constantly establish connections between elements while organizing language. Such complex processing posts high demand on executive functions such as selective attention, working memory, inhibition, planning, and cognitive flexibility (Glosser and Desser 1992; Kintz et al. 2016). Consequently, older adults with reduced domain general cognitive ability might experience more difficulties with sequential picture description tasks compared to single picture or procedural discourses. In summary, accurately evaluating an individual's language proficiency in discourse production requires implementing multiple elicitation methods. Examiners should strategically select appropriate genres based on their assessment objectives. For instance, if the goal is to assess language proficiency without bias from cognitive abilities, genres with lower cognitive demands can be chosen. Conversely, if the aim is to determine whether language impairment is associated with cognitive decline, a combination of genres with both high and low cognitive demands can be utilized for comparison.

This study has several limitations. First, this study exclusively focuses on descriptive discourse, other genres, such as narratives, also merit similar normative data. Moreover, we did not incorporate multiple stimuli for each of the genres we examined (e.g., only one task was used for procedural description), which might limit the thorough exploration of genre‐specific linguistic characteristics, as well as the impact of age and education. Future studies should aim to create a more expansive, multi‐level normative reference for other discourse genres. Second, the scoring of macro‐structural variables might be subject to reliability issues and subjectivity. Although the ICC analysis revealed excellent inter‐rater reliability, potential subjectivity and rater inconsistency may still arise, as the scoring process of ICU‐ and MC‐based measures may inevitably involve subjective decisions of alternative forms. Third, it is yet to be determined if the examined connected speech measures maintain their stability when speech samples are collected at multiple time points. The test‐retest reliability issue may affect its application in research and clinical settings as an outcome measure for language change. Subsequent research should delve deeper into how these newly established normative data can be employed to monitor treatment progress in clinical practice. Finally, as pointed out earlier, the choice of certain connected speech measures might not be optimal to depict genuine linguistic characteristics of the individual; for instance, TTR has limited capacity to reflect lexical diversity when the length of the samples has high variation. Future studies should consider using adjusted measures or different elicitation methods to gather more indicative normative data.

To conclude, this study has established one of a few comprehensive, multi‐level, large‐scale normative references for connected speech measures in the Cantonese‐speaking population. By embracing an extensive range of connected speech measures, systematic comparison of age and educational subgroups, and exploring genre‐specific characteristics, the study offers a robust tool for systematic and objective assessment of connected speech production. The normative data developed serves not only as a valuable diagnostic tool for diseased population like aphasia but also as an outcome measure for monitoring treatment progress in clinical and research settings. The study, however, acknowledges limitations, such as the limited focus on descriptive discourse, potential inter‐rater and test‐retest reliability issues, and the restricted selection of connected speech measures. Future research should aim to supplement this work by exploring other discourse genres, implementing multiple raters for scoring, testing the test‐retest reliability, and considering adjusted measures to accommodate sample length variations. Nevertheless, the current study contributes a significant step towards a more comprehensive understanding and evaluation of connected speech, providing a foundation for future studies and clinical applications.

## Conflicts of Interest

The authors declare no conflicts of interest.

## Supporting information



Supporting Information

## Data Availability

The data that support the findings of this study are available from the corresponding author, AK, upon reasonable request.

## References

[jlcd70055-bib-0001] Arbuckle, T. Y. , M. Nohara‐LeClair , and D. Pushkar .2000. “Effect of Off‐Target Verbosity on Communication Efficiency in a Referential Communication Task.” Psychology and Aging 15, no. 1: 65–77. 10755290 10.1037//0882-7974.15.1.65

[jlcd70055-bib-0074] Bartko J. J. . 1966. “The intraclass correlation coefficient as a measure of reliability.” Psychological Reports 19, no. 1: 3–11. 10.2466/pr0.1966.19.1.3 5942109

[jlcd70055-bib-0002] Baddeley, A. D. 1996. “Exploring the Central Executive.” Quarterly Journal of Experimental Psychology 49: 5–28. 10.1080/713755608

[jlcd70055-bib-0003] Berube S. , J. Nonnemacher , C. Demsky , et al. 2019. “Stealing Cookies in the Twenty‐First Century: Measures of Spoken Narrative in Healthy Versus Speakers With Aphasia.” American Journal of speech‐language Pathology 28, no. 1S: 321–329. 30242341 10.1044/2018_AJSLP-17-0131 PMC6437702

[jlcd70055-bib-0004] Berube S. K. , E. Goldberg , S. M. Sheppard , et al. 2022. “An Analysis of Right Hemisphere Stroke Discourse in the Modern Cookie Theft Picture.” American Journal of speech‐language Pathology 31, no. 5S: 2301–2312. 36075208 10.1044/2022_AJSLP-21-00294 PMC9907448

[jlcd70055-bib-0005] Boucher J. , A. Brisebois , A. Slegers , et al. 2022. “Picture Description of the Western Aphasia Battery Picnic Scene: Reference Data for the French Canadian Population.” American Journal of Speech‐Language Pathology 31, no. 1: 257–270. 34735273 10.1044/2021_AJSLP-20-00388

[jlcd70055-bib-0006] Boyle, M. 2020. “Choosing Discourse Outcome Measures to Assess Clinical Change.” In Seminars in Speech and Language, vol. 41, 1–9. Thieme Medical Publishers. 31869844 10.1055/s-0039-3401029

[jlcd70055-bib-0007] Brookshire, R. H. , and L. E. Nicholas . 1993. “Test‐Retest Stability of Measures of Connected Speech in Aphasia.” Clinical Aphasiology 22: 119–133.

[jlcd70055-bib-0008] Bryant, L. , A. Ferguson , and E. Spencer . 2016. “Linguistic Analysis of Discourse in Aphasia: A Review of the Literature.” Clinical Linguistics & Phonetics 30, no. 7: 489–518. 27002416 10.3109/02699206.2016.1145740

[jlcd70055-bib-0009] Capilouto, G. , H. H. Wright , and S. A. Wagovich . 2005. “CIU and Main Event Analyses of the Structured Discourse of Older and Younger Adults.” Journal of Communication Disorders 38, no. 6: 431–444. 16199238 10.1016/j.jcomdis.2005.03.005

[jlcd70055-bib-0010] Capilouto, G. J. , and H. H. Wright . 2009. “Manipulating Task Instructions to Change Narrative Discourse Performance.” Aphasiology 23: 1295–1308.

[jlcd70055-bib-0011] Capilouto G. J. , H. H. Wright , and K. M. Maddy . 2016. “Microlinguistic Processes That Contribute to the Ability to Relay Main Events: Influence of Age.” Aging, Neuropsychology, and Cognition 23, no. 4: 445–463. 10.1080/13825585.2015.1118006 PMC4939436 26653413

[jlcd70055-bib-0012] Choi, H. 2009. “Performances in a Picture Description Task in Japanese Patients With Alzheimer's Disease and With Mild Cognitive Impairment.” Communication Sciences & Disorders 14, no. 3: 326–337.

[jlcd70055-bib-0013] Cooper, P. V. 1990. “Discourse Production and Normal Aging: Performance on Oral Picture Description Tasks.” Journal of Gerontology 45, no. 5: P210–P214. 2394918 10.1093/geronj/45.5.p210

[jlcd70055-bib-0014] Cotrena, C. , L. D. Branco , C. O. Cardoso , C. E. I. Wong , and R. P. Fonseca . 2016. “The Predictive Impact of Biological and Sociocultural Factors On Executive Processing: The Role of Age, Education, and Frequency of Reading and Writing Habits.” Applied Neuropsychology: Adult 23: 75–84. 10.1080/23279095.2015.1012760 26111081

[jlcd70055-bib-0015] Covington, M. A. , and J. D. McFall . 2010. “Cutting the Gordian Knot: The Moving‐Average Type–Token Ratio (MATTR).” Journal of Quantitative Linguistics 17, no. 2: 94–100.

[jlcd70055-bib-0016] Craik, F. I. , and N. S. Rose . 2012. “Memory Encoding and Aging: A Neurocognitive Perspective.” Neuroscience & Biobehavioral Reviews 36, no. 7: 1729–1739. 22155274 10.1016/j.neubiorev.2011.11.007

[jlcd70055-bib-0017] Dixon, W. J. 1980. “Efficient Analysis of Experimental Observations.” Annual Review of Pharmacology and Toxicology 20, no. 1: 441–462. 10.1146/annurev.pa.20.040180.002301 7387124

[jlcd70055-bib-0018] Duong, A. , and B. Ska . 2001. “Production of Narratives: Picture Sequence Facilitates Organizational but Not Conceptual Processing in Less Educated Subjects.” Brain and Language 45, no. 121: 124. 10.1016/s0278-2626(01)80047-6 11527309

[jlcd70055-bib-0019] Fergadiotis, G. , H. H. Wright , and G. J. Capilouto . 2011. “Productive Vocabulary Across Discourse Types.” Aphasiology 25, no. 10: 1261–1278. 10.1080/02687038.2011.606974 22904592 PMC3419587

[jlcd70055-bib-0020] Giles, E. , K. Patterson , and J. R. Hodges . 1996. “Performance on the Boston Cookie Theft Picture Description Task in Patients With Early Dementia of the Alzheimer's Type: Missing Information.” Aphasiology 10, no. 4: 395–408.

[jlcd70055-bib-0021] Glosser, G. , and T. Deser . 1992. “A Comparison of Changes in Macrolinguistic and Microlinguistic Aspects of Discourse Production in Normal Aging.” Journal of Gerontology 47, no. 4: P266–P272. 1624704 10.1093/geronj/47.4.p266

[jlcd70055-bib-0022] Goodglass, H. , and E. Kaplan . 1993. The Assessment of Aphasia and Related Disorders. Lea & Febiger.

[jlcd70055-bib-0023] Goodglass, H. , E. Kaplan , and B. Barresi . 2001. BDAE: the Boston Diagnostic Aphasia Examination. Lippincott Williams & Wilkins.

[jlcd70055-bib-0024] Graesser, A. C. , D. S. McNamara , M. M. Louwerse , and Z. Cai . 2004. “Coh‐Metrix: Analysis of Text on Cohesion and Language.” Behavior Research Methods, Instruments, & Computers 36, no. 2: 193–202. 10.3758/bf03195564 15354684

[jlcd70055-bib-0025] Herbert, R. , J. Hickin , D. Howard , F. Osborne , and W. Best . 2008. “Do Picture‐Naming Tests Provide a Valid Assessment of Lexical Retrieval in Conversation in Aphasia?” Aphasiology 22, no. 2: 184–203.

[jlcd70055-bib-0026] Hulit, L. M. , M. R. Howard , and K. R. Fahey . 2011 Born to Talk: an Introduction to Speech and Language Development. Pearson.

[jlcd70055-bib-0027] Hux, K. , and K. Frodsham . 2023. “Speech and Language Characteristics of Neurologically Healthy Adults When Describing the Modern Cookie Theft Picture: Mixing the New with the Old.” American Journal of Speech‐Language Pathology 32, no. 3: 1110–1130. 36898138 10.1044/2022_AJSLP-22-00291

[jlcd70055-bib-0028] James, L. E. , D. M. Burke , A. Austin , and E. Hulme . 1998. “Production and Perception of ‘Verbosity’ in Younger and Older Adults.” Psychology and Aging 13, no. 3: 355–367. 9793112 10.1037//0882-7974.13.3.355

[jlcd70055-bib-0029] Jensen, A. M. , H. J. Chenery , and D. A. Copland . 2006. “A Comparison of Picture Description Abilities in Individuals With Vascular Subcortical Lesions and Huntington's Disease.” Journal of Communication Disorders 39, no. 1: 62–77. 16154582 10.1016/j.jcomdis.2005.07.001

[jlcd70055-bib-0030] Juncos‐Rabadán, O. 1996. “Narrative Speech in the Elderly: Effects of Age and Education On Telling Stories.” International Journal of Behavioral Development 19: 669–685. 10.1177/016502549601900313

[jlcd70055-bib-0031] Juncos‐Rabadán, O. , A. X. Pereiro , and M. S. Rodríguez . 2005. “Narrative Speech in Aging: Quantity, Information Content, and Cohesion.” Brain and Language 95, no. 3: 423–434. 15913755 10.1016/j.bandl.2005.04.001

[jlcd70055-bib-0033] Kavé G. , and Y. Levy . 2003. “Morphology in Picture Descriptions Provided by Persons With Alzheimer's Disease.” Journal of Speech, Language, and Hearing Research 46, no. 2: 341–352. 10.1044/1092-4388(2003/027) 14700376

[jlcd70055-bib-0034] Kertesz, A. 2006. Western Aphasia Battery‐Revised (WAB‐R). Pro‐Ed.

[jlcd70055-bib-0071] Kertesz, A. 1982. Western Aphasia Battery. Pro‐Ed.

[jlcd70055-bib-0035] Kintsch, W. , and T. A. van Dijk . 1978. “Toward a Model of Text Comprehension and Production.” Psychological Review 85, no. 5: 363–394. 10.1037/0033-295X.85.5.363

[jlcd70055-bib-0036] Kintz, S. , G. Fergadiotis , and H. H. Wright . 2016. “Aging Effects on Discourse Production.” In Cognition, Language and Aging, edited by H. H. Wright , 81–106. John Benjamins Publishing Company.

[jlcd70055-bib-0037] Kong, A. P. H. 2009. “The Use of Main Concept Analysis to Measure Discourse Production in Cantonese‐Speaking Persons With Aphasia: A Preliminary Report.” Journal of Communication Disorders 42, no. 6: 442–464. 19643430 10.1016/j.jcomdis.2009.06.002

[jlcd70055-bib-0072] Kong, A. P. H. 2011. “The main concept analysis in Cantonese aphasic oral discourse: External validation and monitoring chronic aphasia.” Journal of Speech, Language, and Hearing Research 54: 148–159. 10.1044/1092-4388(2010/09-0240) 20719865

[jlcd70055-bib-0038] Kong, A. P. H. 2022. “Clinical Assessment of Disordered Discourse.” In Analysis of Neurogenic Disordered Discourse Production: Theories, Assessment and Treatment, edited by A. P. H. Kong , 79–96. Psychology Press.

[jlcd70055-bib-0039] Kong, A. P. H. , and S. P. Law . 2019. “Cantonese AphasiaBank: An Annotated Database of Spoken Discourse and Co‐Verbal Gestures by Healthy and Language‐Impaired Native Cantonese Speakers.” Behavior Research Methods 51, no. 3: 1131–1144. 10.3758/s13428-018-1043-6 29693232 PMC6200664

[jlcd70055-bib-0040] Kong, A. P. H. , and C. W. Y. Wong . 2018. “An Integrative Analysis of Spontaneous Storytelling Discourse in Aphasia: Relationship with Listeners' Rating and Prediction of Severity and Fluency Status of Aphasia.” American Journal of Speech‐Language Pathology 27, no. 4: 1491–1505. 30458505 10.1044/2018_AJSLP-18-0015 PMC6436460

[jlcd70055-bib-0041] Le Dorze, G. , and C. Bédard . 1998. “Effects of Age and Education on the Lexico‐Semantic Content of Connected Speech in Adults.” Journal of Communication Disorders 31, no. 1: 53–71. 10.1016/S0021-9924(97)00051-8. 9421767

[jlcd70055-bib-0042] Lilliefors, H. W. 1967. “On the Kolmogorov‐Smirnov Test for Normality With Mean and Variance Unknown.” Journal of the American Statistical Association 62, no. 318: 399–402.

[jlcd70055-bib-0043] Mackenzie, C. 2000. “Adult Spoken Discourse: The Influences of Age and Education.” International Journal of Language & Communication Disorders 35, no. 2: 269–285. 10912255 10.1080/136828200247188

[jlcd70055-bib-0044] Macwhinney, B. 2000. The CHILDES Project: Tools for Analyzing Talk, vol. 1. Psychology Press.

[jlcd70055-bib-0073] Malcorra, B. L. C. , M. A. Wilson , L. P. Schilling , and L. C. Hübner . 2022. “Lower Education and Reading and Writing Habits Are Associated With Poorer Oral Discourse Production in Typical Adults and Older Adults.” Frontiers in Psychology 13: 740337. 10.3389/fpsyg.2022.740337 35369132 PMC8972065

[jlcd70055-bib-0045] Marini, A. , A. Boewe , C. Caltagirone , and S. Carlomagno . 2005. “Age‐Related Differences in the Production of Textual Descriptions.” Journal of Psycholinguistic Research 34: 439–463. 16177935 10.1007/s10936-005-6203-z

[jlcd70055-bib-0046] Mayer, J. , and L. Murray . 2003. “Functional Measures of Naming in Aphasia: Word Retrieval in Confrontation Naming versus Connected Speech.” Aphasiology 17, no. 5: 481–497.

[jlcd70055-bib-0047] McCarthy, P. M. , and S. Jarvis . 2010. “MTLD, Vocd‐D, and HD‐D: A Validation Study of Sophisticated Approaches to Lexical Diversity Assessment.” Behavior Research Methods 42, no. 2: 381–392. 20479170 10.3758/BRM.42.2.381

[jlcd70055-bib-0048] Mckee G. 2000. “Measuring Vocabulary Diversity Using Dedicated Software.” Literary and Linguistic Computing 15, no. 3: 323–338. 10.1093/llc/15.3.323.

[jlcd70055-bib-0049] Menn, L. , G. Ramsberger , and N. Helm‐Estabrooks . 1994. “A Linguistic Communication Measures for Aphasic Narratives.” Aphasiology 8, no. 4: 343–359.

[jlcd70055-bib-0050] Mulder, K. , and J. H. Hulstijn . 2011. “Linguistic Skills of Adult Native Speakers, as a Function of Age and Level of Education.” Applied Linguistics 32, no. 5: 475–494.

[jlcd70055-bib-0051] Nicholas, L. E. , and R. H. Brookshire . 1995. “Presence, Completeness, and Accuracy of Main Concepts in the Connected Speech of Non‐Brain‐Damaged Adults and Adults With Aphasia.” Journal of Speech and Hearing Research 38: 145–156. 7731205 10.1044/jshr.3801.145

[jlcd70055-bib-0052] Nicolas, L. E. , and R. H. Brookshire . 1993. “A System for Quantifying the Informativeness and Efficiency of the Connected Speech of Adults With Aphasia.” Journal of Speech and Hearing Research 36: 338–350. 8487525 10.1044/jshr.3602.338

[jlcd70055-bib-0053] North, A. J. , H. K. Ulatowska , S. Macaluso‐Haynes , and H. Bell . 1986. “Discourse Performance in Older Adults.” International Journal of Aging and Human Development 23, no. 4: 267–283. 3557641 10.2190/BPF0-2BWD-BGNQ-HWCW

[jlcd70055-bib-0054] Potechin, G. G. , L. E. Nicholas , and R. H. Brookshire . 1987. “Effects of Picture Stimuli on Discourse Production.” Aphasiology 17: 216–220.

[jlcd70055-bib-0055] Pritchard, M. , L. Dipper , G. Morgan , and N. Cocks . 2015. “Language and Iconic Gesture Use in Procedural Discourse by Speakers With Aphasia.” Aphasiology 29, no. 7: 826–844. 25999636 10.1080/02687038.2014.993912 PMC4409036

[jlcd70055-bib-0056] Pushkar, D. , P. Basevitz , T. Arbuckle , M. Nohara‐LeClair , S. Lapidus , and M. Peled . 2000. “Social Behavior and Off‐Target Verbosity in Elderly People.” Psychology and Aging 15, no. 2: 361–374. 10879589 10.1037//0882-7974.15.2.361

[jlcd70055-bib-0057] Richardson, J. D. , and S. G. Dalton . 2016. “Main Concepts for Three Different Discourse Tasks in a Large Non‐Clinical Sample.” Aphasiology 30, no. 1: 45–73. 40012631 10.1080/02687038.2015.1057891 PMC11864783

[jlcd70055-bib-0058] Schuell, H. 1965. Minnesota Test for Differential Diagnosis of Aphasia. University of Minnesota Press. 10.1044/jshr.0504.349 13987135

[jlcd70055-bib-0059] Small, S. A. , Y. Stern , M. Tang , and R. Mayeux . 1999. “Selective Decline in Memory Function Among Healthy Elderly.” Neurology 52: 1392–1996. 10.1212/WNL.52.7.1392. 10227623

[jlcd70055-bib-0060] Taler, V. , and N. A. Phillips . 2008. “Language Performance in Alzheimer's Disease and Mild Cognitive Impairment: A Comparative Review.” Journal of Clinical and Experimental Neuropsychology 30, no. 5: 501–556. 18569251 10.1080/13803390701550128

[jlcd70055-bib-0061] Templin, M. C. 1957. Certain Language Skills in Children: Their Development and Interrelationships, vol. 10. University of Minnesota Press.

[jlcd70055-bib-0062] Tomarken, A. J. , and R. C. Serlin . 1986. “Comparison of ANOVA Alternatives Under Variance Heterogeneity and Specific Noncentrality Structures.” Psychological Bulletin 99, no. 1: 90–99.

[jlcd70055-bib-0063] Ulatowska, H. , G. Streit Olness , R. Wertz , A. Samson , M. Keebler , and K. Goins . 2003. “Relationship Between Discourse and Western Aphasia Battery Performance in African Americans With Aphasia.” Aphasiology 17, no. 5: 511–521. 10.1080/0268703034400102 PMC12383048 40881852

[jlcd70055-bib-0064] Ulatowska, H. K. , and G. S. Olness . 2004. “Discourse.” In The MIT Encyclopedia of Communication Disorders, edited by R. Kent , 300–302. The MIT Press.

[jlcd70055-bib-0065] Weisstein, E. W. 2004. Bonferroni Correction . https://mathworld.wolfram.com/.

[jlcd70055-bib-0066] Williams, C. , A. Thwaites , P. Buttery , et al. 2010. “The Cambridge Cookie‐Theft Corpus: A Corpus of Directed and Spontaneous Speech of Brain‐Damaged Patients and Healthy Individuals.” In LREC, edited by N. Calzolari , K. Choukri , B. Maegaard , B. Maegaard , J. Mariani , J. Odijk , S. Piperidis , M. Rosner , and D. Tapias , 2824–2830. European Language Resources Association (ELRA).

[jlcd70055-bib-0067] Wright, H. H. , G. J. Capilouto , S. A. Wagovich , T. Cranfill , and J. E. Davis . 2005. “Development and Reliability of a Quantitative Measure of Adults' Narratives.” Aphasiology 19, no. 3/4/5: 263–273. 10.1080/02687030444000732.

[jlcd70055-bib-0068] Wright, H. H. , A. D. Koutsoftas , G. J. Capilouto , and G. Fergadiotis . 2014. “Global Coherence in Younger and Older Adults: Influence of Cognitive Processes and Discourse Type.” Aging, Neuropsychology, and Cognition 21, no. 2: 174–196. 10.1080/13825585.2013.794894 PMC3748174 23656430

[jlcd70055-bib-0069] Yang, J. S. , C. Rosvold , and N. Bernstein Ratner . 2022. “Measurement of Lexical Diversity in Children's Spoken Language: Computational and Conceptual Considerations.” Frontiers in Psychology 13: 905789. 10.3389/fpsyg.2022.905789 35814069 PMC9257278

