# Supplementary material for: Establishing Norm of Connected Speech Measures for Descriptive Discourses in Cantonese‐Speaking Adults
Source: Int J Lang Commun Disord. 2025 May 19;60(3):e70055. doi: 10.1111/1460-6984.70055 (PMC12086505; doi:10.1111/1460-6984.70055)
Supplement: Supplementary file 1 — Supporting Information [file JLCD-60-0-s001.docx]

Supplementary Table 1. Descriptive statistics of connected speech measures of the real-photo single-picture description - “*Flood*”.

| Variables | Mean(SD) | Median | Minimum | Maximum | Interquartile range | Skewness | Kurtosis | Normality^a^ |
| --- | --- | --- | --- | --- | --- | --- | --- | --- |
| Micro-structural | | | | | | | | |
| Duration (sec) | 37.74(17.34) | 33.00 | 17.75 | 81.00 | 22.50 | 1.084 | .387 | <.001 |
| #Utterances | 11.66(6.49) | 10.00 | 4.50 | 28.50 | 8.00 | 1.216 | .753 | <.001 |
| MLU-w | 7.49(1.50) | 7.33 | 4.88 | 10.33 | 2.21 | .183 | -.779 | .200 |
| Types | 49.58(18.66) | 44.00 | 26.00 | 93.50 | 26.50 | .831 | -.236 | <.001 |
| Tokens | 85.99(47.34) | 69.00 | 30.50 | 204.50 | 62.50 | 1.092 | .350 | <.001 |
| Token/min | 135.75(32.63) | 137.37 | 66.36 | 186.93 | 49.32 | -.334 | -.654 | .200 |
| Verbs/utterance | 1.49(0.38) | 1.43 | 0.90 | 2.28 | 0.57 | .456 | -.637 | .003 |
| Noun-verb ratio | 0.78(0.25) | 0.73 | 0.39 | 1.43 | 0.29 | .944 | .768 | .002 |
| O-C ratio | 1.04(0.25) | 1.00 | 0.64 | 1.62 | 0.40 | .470 | -.656 | .023 |
| TTR | 0.63(0.11) | 0.62 | 0.44 | 0.82 | 0.16 | -.030 | -.875 | .200 |
| Retracing | 0.52(0.74) | 0.00 | 0.00 | 2.00 | 1.00 | 1.054 | -.375 | <.001 |
| Repetitions | 1.25(1.48) | 1.00 | 0.00 | 5.00 | 2.00 | 1.150 | .426 | <.001 |
| Macro-structural | | | | | | | | |
| Total ICUs | 9.85(2.74) | 10.00 | 5.00 | 15.00 | 4.00 | .032 | -.631 | .006 |
| ICUs/sec | 0.31(0.14) | 0.29 | 0.08 | 0.59 | 0.22 | .406 | -.728 | .023 |
| ICUs/word | 0.14(0.07) | 0.13 | 0.04 | 0.31 | 0.10 | .681 | -.127 | .035 |
| ICUs/utterance | 1.05(0.49) | 1.00 | 0.31 | 2.00 | 0.80 | .276 | -.967 | .003 |
| MC scores | 9.34(2.21) | 9.00 | 4.00 | 12.00 | 3.00 | -.560 | -.013 | <.001 |
| ACs/min | 5.81(2.74) | 5.45 | 1.39 | 11.08 | 4.38 | .294 | -.903 | .053 |

*Note.* ^a^Normality verified by Kolmogorov-Smirov test, *p* < .05 infers non-normally distributed data.

Supplementary Table 2. Descriptive statistics of connected speech measures of the line-drawing single-picture description - “*Cat rescue*”.

| Variables | Mean(SD) | Median | Minimum | Maximum | Interquartile range | Skewness | Kurtosis | Normality^a^ |
| --- | --- | --- | --- | --- | --- | --- | --- | --- |
| Micro-structural | | | | | | | | |
| Duration (sec) | 48.10(21.12) | 40.00 | 24.00 | 102.50 | 26.00 | 1.228 | .817 | <.001 |
| #Utterances | 17.27(8.24) | 15.00 | 7.00 | 35.00 | 12.00 | .734 | -.455 | <.001 |
| MLU-w | 7.47(1.33) | 7.33 | 5.38 | 10.44 | 1.97 | .556 | -.307 | .004 |
| Types | 62.50(18.17) | 60.00 | 36.50 | 101.50 | 25.50 | .595 | -.460 | .016 |
| Tokens | 124.70(53.67) | 114.00 | 56.00 | 244.00 | 84.00 | .703 | -.485 | <.001 |
| Token/min | 159.41(28.79) | 161.45 | 105.98 | 208.30 | 43.16 | -.191 | -.854 | .023 |
| Verbs/utterance | 1.39(0.30) | 1.34 | 0.91 | 2.06 | 0.40 | .496 | -.382 | .055 |
| Noun-verb ratio | 0.85(0.18) | 0.83 | 0.56 | 1.23 | 0.26 | .469 | -.561 | .002 |
| O-C ratio | 1.08(0.26) | 1.03 | 0.76 | 1.75 | 0.34 | 1.039 | .587 | <.001 |
| TTR | 0.53(0.09) | 0.53 | 0.38 | 0.68 | 0.16 | -.057 | -1.199 | .034 |
| Retracing | 0.81(0.95) | 1.00 | 0.00 | 3.00 | 1.00 | .906 | -.263 | <.001 |
| Repetitions | 1.03(1.29) | 1.00 | 0.00 | 4.50 | 2.00 | 1.328 | .971 | <.001 |
|  | | | | | | | | |
| Total ICUs | 27.01(6.25) | 27.00 | 16.00 | 38.00 | 9.00 | -.037 | -.807 | .047 |
| ICUs/sec | 0.62(0.19) | 0.60 | 0.30 | 1.02 | 0.27 | .317 | -.626 | .200 |
| ICUs/word | 0.24(0.07) | 0.24 | 0.12 | 0.39 | 0.11 | .249 | -.749 | .200 |
| ICUs/utterance | 1.81(0.66) | 1.73 | 0.82 | 3.17 | 1.02 | .392 | -.733 | .049 |
| MC scores | 24.38(4.01) | 24.00 | 15.50 | 30.00 | 5.50 | -.533 | -.413 | <.001 |
| ACs/min | 11.36(3.75) | 10.71 | 5.27 | 18.69 | 4.98 | .322 | -.699 | .012 |

*Note.* ^a^Normality verified by Kolmogorov-Smirov test, *p* < .05 infers non-normally distributed data.

Supplementary Table 3. Descriptive statistics of connected speech measures of the sequential 4-picture description - “*Broken window*”.

| Variables | Mean(SD) | Median | Minimum | Maximum | Interquartile range | Skewness | Kurtosis | Normality^a^ |
| --- | --- | --- | --- | --- | --- | --- | --- | --- |
| Micro-structural | | | | | | | | |
| Duration (sec) | 33.61(13.19) | 29.00 | 16.50 | 66.50 | 16.00 | .984 | .303 | <.001 |
| #Utterances | 11.47(5.37) | 10.00 | 5.00 | 25.50 | 6.00 | 1.176 | .781 | <.001 |
| MLU-w | 7.17(1.30) | 7.08 | 5.13 | 10.25 | 1.73 | .580 | .031 | .010 |
| Types | 46.31(15.01) | 45.00 | 24.50 | 81.00 | 18.00 | .749 | -.054 | <.001 |
| Tokens | 81.73(37.62) | 73.00 | 36.00 | 180.50 | 41.00 | 1.099 | .618 | <.001 |
| Token/min | 145.79(30.88) | 146.67 | 81.96 | 195.59 | 47.58 | -.223 | -.707 | .200 |
| Verbs/utterance | 1.30(0.30) | 1.29 | 0.86 | 2.00 | 0.38 | .651 | -.119 | .087 |
| Noun-verb ratio | 1.07(0.27) | 1.00 | 0.64 | 1.65 | 0.42 | .370 | -.638 | <.001 |
| O-C ratio | 1.03(0.23) | 1.00 | 0.70 | 1.51 | 0.33 | .501 | -.634 | .005 |
| TTR | 0.60(0.09) | 0.61 | 0.44 | 0.76 | 0.13 | -.025 | -.776 | .200 |
| Retracing | 0.62(0.90) | 0.00 | 0.00 | 3.00 | 1.00 | 1.388 | 1.031 | <.001 |
| Repetitions | 0.45(0.70) | 0.00 | 0.00 | 2.00 | 1.00 | 1.252 | .157 | <.001 |
|  | | | | | | | | |
| Total ICUs | 17.15(4.51) | 17.00 | 8.50 | 24.50 | 7.00 | -.259 | -.812 | <.001 |
| ICUs/sec | 0.56(0.20) | 0.58 | 0.24 | 0.92 | 0.30 | .049 | -.966 | .200 |
| ICUs/word | 0.24(0.08) | 0.23 | 0.09 | 0.38 | 0.13 | -.004 | -1.046 | .020 |
| ICUs/utterance | 1.70(0.61) | 1.75 | 0.65 | 2.82 | 0.98 | .060 | -.972 | .017 |
| MC scores | 18.53(3.89) | 18.00 | 11.00 | 24.00 | 5.00 | -.333 | -.797 | <.001 |
| ACs/min | 11.58(4.44) | 11.67 | 3.34 | 20.43 | 6.21 | .132 | -.542 | .200 |

*Note.* ^a^Normality verified by Kolmogorov-Smirov test, *p* < .05 infers non-normally distributed data.

Supplementary Table 4. Descriptive statistics of connected speech measures of the sequential 6-picture description - “*Refuse umbrella*”.

| Variables | Mean(SD) | Median | Minimum | Maximum | Interquartile range | Skewness | Kurtosis | Normality^a^ |
| --- | --- | --- | --- | --- | --- | --- | --- | --- |
| Micro-structural | | | | | | | | |
| Duration (sec) | 36.09(10.37) | 35.00 | 21.00 | 58.50 | 15.00 | .585 | -.466 | <.001 |
| #Utterances | 16.73(6.40) | 15.00 | 8.00 | 30.50 | 9.00 | .615 | -.627 | <.001 |
| MLU-w | 6.10(0.92) | 6.00 | 4.64 | 7.93 | 1.23 | .400 | -.616 | .015 |
| Types | 55.96(14.44) | 54.00 | 34.00 | 86.50 | 20.50 | .473 | -.532 | .013 |
| Tokens | 100.14(35.11) | 92.00 | 52.00 | 178.00 | 53.50 | .665 | -.456 | <.001 |
| Token/min | 166.00(28.52) | 166.53 | 113.44 | 214.98 | 43.63 | -.102 | -.830 | .200 |
| Verbs/utterance | 1.30(0.28) | 1.23 | 0.90 | 1.90 | 0.41 | .586 | -.518 | <.001 |
| Noun-verb ratio | 0.74(0.19) | 0.73 | 0.42 | 1.16 | 0.26 | .229 | -.483 | .200 |
| O-C ratio | 1.42(0.39) | 1.35 | 0.91 | 2.26 | 0.55 | .738 | -.431 | <.001 |
| TTR | 0.58(0.08) | 0.57 | 0.45 | 0.75 | 0.13 | .379 | -.784 | .040 |
| Retracing | 0.38(0.64) | 0.00 | 0.00 | 2.00 | 1.00 | 1.454 | .878 | <.001 |
| Repetitions | 0.45(0.72) | 0.00 | 0.00 | 2.00 | 1.00 | 1.276 | .125 | <.001 |
|  | | | | | | | | |
| Total ICUs | 25.76(4.88) | 26.00 | 16.00 | 34.50 | 7.00 | -.175 | -.602 | .200 |
| ICUs/sec | 0.75(0.19) | 0.74 | 0.45 | 1.14 | 0.26 | .267 | -.676 | .200 |
| ICUs/word | 0.28(0.07) | 0.27 | 0.16 | 0.42 | 0.11 | .288 | -.835 | .200 |
| ICUs/utterance | 1.69(0.51) | 1.64 | 0.79 | 2.63 | 0.77 | .181 | -.780 | .050 |
| MC scores | 30.06(3.23) | 30.00 | 22.50 | 33.00 | 4.50 | -.950 | -.126 | <.001 |
| ACs/min | 17.48(5.10) | 16.88 | 8.63 | 28.10 | 7.13 | .304 | -.589 | .082 |

*Note.* ^a^Normality verified by Kolmogorov-Smirov test, *p* < .05 infers non-normally distributed data.

Supplementary Table 5. Descriptive statistics of connected speech measures of the procedural description - “*Egg ham sandwich*”.

| Variables | Mean(SD) | Median | Minimum | Maximum | Interquartile range | Skewness | Kurtosis | Normality^a^ |
| --- | --- | --- | --- | --- | --- | --- | --- | --- |
| Micro-structural | | | | | | | | |
| Duration (sec) | 33.22(14.71) | 31.00 | 14.00 | 65.50 | 20.00 | .725 | -.399 | <.001 |
| #Utterances | 12.09(6.04) | 10.00 | 5.00 | 26.00 | 9.00 | .771 | -.348 | <.001 |
| MLU-w | 6.64(1.24) | 6.56 | 4.53 | 9.32 | 1.67 | .348 | -.336 | .200 |
| Types | 44.42(16.40) | 41.00 | 22.00 | 79.50 | 25.00 | .502 | -.693 | <.001 |
| Tokens | 80.85(42.41) | 71.00 | 26.50 | 177.50 | 63.50 | .745 | -.291 | <.001 |
| Token/min | 141.92(30.66) | 143.20 | 85.89 | 195.69 | 52.52 | -.115 | -.933 | .200 |
| Verbs/utterance | 1.14(0.28) | 1.07 | 0.79 | 1.80 | 0.34 | .984 | .200 | <.001 |
| Noun-verb ratio | 1.01(0.27) | 1.00 | 0.54 | 1.50 | 0.42 | .034 | -.901 | .200 |
| O-C ratio | 1.25(0.49) | 1.06 | 0.70 | 2.59 | 0.52 | 1.322 | 1.125 | <.001 |
| TTR | 0.60(0.12) | 0.59 | 0.43 | 0.86 | 0.16 | .564 | -.462 | .003 |
| Retracing | 0.45(0.68) | 0.00 | 0.00 | 2.00 | 1.00 | 1.220 | .176 | <.001 |
| Repetitions | 0.55(0.78) | 0.00 | 0.00 | 2.50 | 1.00 | 1.181 | .164 | <.001 |
|  | | | | | | | | |
| Total ICUs | 16.47(5.68) | 16.00 | 7.50 | 28.00 | 8.50 | .295 | -.738 | .030 |
| ICUs/sec | 0.54(0.17) | 0.52 | 0.28 | 0.92 | 0.24 | .416 | -.385 | .200 |
| ICUs/word | 0.24(0.09) | 0.23 | 0.11 | 0.42 | 0.13 | .440 | -.752 | .040 |
| ICUs/utterance | 1.54(0.56) | 1.50 | 0.70 | 2.60 | 0.80 | .289 | -.895 | .066 |
| MC scores | 10.05(2.16) | 10.00 | 6.00 | 12.00 | 3.00 | -.647 | -.837 | <.001 |
| ACs/min | 6.94(2.87) | 6.49 | 2.70 | 13.33 | 3.67 | .635 | -.208 | .008 |

*Note.* ^a^Normality verified by Kolmogorov-Smirov test, *p* < .05 infers non-normally distributed data.

Supplementary Table 6. Standard information content units (ICUs) for the real-photo single-picture description - “*Flood*”.

| Scenario | ICUs (frequency) | | | | |
| --- | --- | --- | --- | --- | --- |
|  | Subjects | Places | Objects | Actions | Others |
| Cantonese version | | | | | |
| 1. Setting | 1. **女仔/女人/女/妹妹/小朋友1** [-去] (31.33%) | 1. **河/激流/溪澗/海** (20.67%) | 1. **河水/洪水** [-泛濫] (21.33%) | 1. [女仔-] **去/玩/行/行山/遠足/旅行/划艇** (32.67%) | 1. [發生-] **水災/暴雨/海嘯/颱風/水浸/山洪暴發** (27.33%) |
| 1. The girl falls into the river | 1. **女仔2** [-跌] (70.00%) | 1. **水/河/急流/激流/溪澗/海/湖/洪水** (64.00%) | 1. [捉-] **樹/樹枝/樹幹/木/竹1** (23.33%) 2. **水/洪水** [-沖] (32.67%) | 1. [女仔-] **跌/跣/跌落** (42.00%) 2. [女仔-] **沖/沖走/漂流** (44.67%) 3. [女仔-] **捉/掹/攬/搲/揸/抓** (22.67%) |  |
| 1. The man rescues the girl | 1. **男人/爸爸/救生員/救護員/消防員/拯救員** [-救] (88.67%) 2. [救-] **女仔3** (85.33%) | **/** | 1. [捉-] **樹2** (44.67%) 2. [著-] **救生衣** | 1. [男人-] **嚟/去/落/衝/趕到/落去** (47.33%) 2. [男人-] **想/諗住/嘗試/意圖/希望** (26.67%) 3. [男人-] **救/搶救/拯救** (86.00%) 4. [男人-] **掹/捉/挨/扶/攬/揸/扯** (35.33%) 5. [男人-] **著** (30.67%) | 1. [救-] **上/上嚟/返嚟/出嚟** (24.67%) |
| English version | | | | | |
| 1. Setting | 1. **Girl /Woman/ Female/Sister/ Child 1** [-go] (31.33%) | 1. **River/Torrent/ Stream/Sea** (20.67%) | 1. **River/Flood** [-overflow] (21.33%) | 1. [Girl-] **Go/Play/ Walk/Hike/Travel/ Row** (32.67%) | 1. [There was a-] **Inundation/Rainstorm/ Tsunami/Typhoon/ Flooding/Freshet** (27.33%) |
| 1. The girl falls into the river | 1. **Girl 2** [-fall] (70.00%) | 1. **Water/River /Torrent/ Stream/Sea/ Lake/Flood** (64.00%) | 1. [Grab-] **Tree/Bough/ Bole/Wood/ Bamboo 1** (23.33%) 2. **Water/Flood** [-Flush] (32.67%) | 1. [Girl-] **Fall/Slip/Fall down** (42.00%) 2. [Girl-] **Flush/Flow** (44.67%) 3. [Girl-] **Grab/Clutch/ Hold//Catch/Snatch** (22.67%) |  |
| 1. The man rescues the girl | 1. **Man/Father/** **Lifeguard/Lifesaver /Fireman/Rescuer** [-save] (88.67%) 2. [Save-] **Girl 3** (85.33%) | **/** | 1. [Grab-] **Tree 2** (44.67%) 2. [Wear-] **Life jacket** | 1. [Man-] **Come/Go/ Get down/Rush** (47.33%) 2. [Man-] **Want/ Wish/Try/ Intend/Hope** (26.67%) 3. [Man-] **Save/Rescue** (86.00%) 4. [Man-] **Grab/Clutch/ Hold//Catch/Snatch** (35.33%) 5. [Man-] **Wear** (30.67%) | 1. [Get her-] **Up/Out/Back** (24.67%) |

Supplementary Table 7. Standard information content units (ICUs) for the line-drawing single-picture description - “*Cat rescue*”.

| Scenario | ICUs (frequency) | | | | |
| --- | --- | --- | --- | --- | --- |
|  | Subjects | Places | Objects | Actions | Others |
| Cantonese version | | | | | |
| 1. Setting | 1. **女仔1/小朋友/阿女** [-玩] (58.67%) 2. **男人1/爸爸/大人/路人** [-玩] (20.67%) 3. **貓1** [-玩] (22.67%) | 1. **公園** (36.00%) | 1. [踩-] **單車/三輪車** (32.67%) | 1. [女仔-] **去/嚟/行/經過** (26.00%) 2. [女仔-] **玩** (34.00%) 3. [女仔-] **踩** (32.00%) | / |
| 1. The girl rescues the cat | 1. **貓2** [-爬] (92.67%) 2. **女仔2** [-叫] (69.33%) 3. [叫-] **貓3** (51.33%) 4. [叫-] **男人2** (28.67%) | 1. **樹1** (86.67%) | / | 1. [貓-] **爬/擒/上/行/跳** (75.33%) 2. [貓-] **落唔到嚟/唔落嚟/**(唔敢)**落嚟1** (38.67%) 3. [女仔-] **見/發現/發覺** (31.33%) 4. [女仔-] **想/諗住** (23.33%) 5. [女仔-] **救/捉/接/抱/攞** (30.00%) 6. [女仔-] **叫1/嗌/氹** (24.00%) 7. [女仔-] **叫2/搵/通知/請求/求救** (29.33%) 8. [貓-] **落嚟2** (36.00%) | 1. [爬-] **上去1/上**   OR [樹-] **上面1** (80.00%) |
| 1. The man stuck on the tree | 1. **男人3** [-爬] (90.67%) 2. [救-] **貓4** (58.00%) 3. **狗** [-追] (84.00%) 4. [-追] **男人4** (41.33%) 5. **男人5** [-落唔到嚟] (39.33%) | 1. **樹2** (76.67%) | 1. [攞-] **梯1** (42.00%) 2. **梯2** [-跌] (30.67%) | 1. [男人-] **攞/擔/用** (40.67%) 2. [男人-] **爬/擒/上/跳/走** (86.00%) 3. [男人-] **想/諗住/嘗試/意圖** (42.00%) 4. [男人-] **幫** (21.33%) 5. [男人-] **救/捉/抱/搦/攞/趕** (50.00%) 6. [貓-] **落嚟3** (27.33%) 7. [梯-] **跌/冧** (32.67%) 8. [狗-] **追/趕/捉** (28.00%) 9. [狗-] **吠/叫** (56.67%) 10. [男人-] **驚/嚇親** (21.33%) 11. [男人-] **落唔到嚟/唔上唔落/**(唔敢)**落嚟2** (56.00%) | 1. **點知** [-狗追男人/梯跌咗] (38.67%) 2. [爬-] **上去2/上**   OR [樹-] **上面2** (75.33%)   1. [樹-] **下面/底** (28.00%) |
| 1. The firemen come | 1. **消防員/消防局** [-救] (92.67%) 2. [-救] **男人6/佢哋** (84.67%) 3. [-救] **貓5** (38.00%) | / | 1. [擔-] **梯3/雲梯** (52.00%) | 1. [男人/女仔-] **打電話/報警** (30.00%) 2. **叫/call/嗌/搵/勞煩/驚動** [-消防員] (62.00%) 3. [消防員-] **嚟/去/過嚟/過去/趕到/到場** (79.33%) 4. [消防員-] **擔/攞/搦/帶** (50.00%) 5. [消防員-] **救/營救/幫/協助** (96.67%) 6. [男人-] **落嚟2** (52.67%) | 1. **最後/結果/卒之** [-消防員嚟] (29.33%) |
| English version | | | | | |
| 1. Setting | 1. **Girl 1/Child/Daughter** [-play] (58.67%) 2. **Man 1/Father/ Adult/Pedestrian** [-play] (20.67%) 3. **Cat 1** [-play] (22.67%) | 1. **Park** (36.00%) | 1. [Ride-] **Bicycle/ Tricycle** (32.67%) | 1. [Girl-] **Go/Come/Walk/Pass** (26.00%) 2. [Girl-] **Play** (34.00%) 3. [Girl-] **Ride** (32.00%) | / |
| 1. The girl rescues the cat | 1. **Cat 2** [-climb] (92.67%) 2. **Girl 2** [-shout] (69.33%) 3. [Shout-] **Cat 3** (51.33%) 4. [Ask-] **Man 2** (28.67%) | 1. **Tree 1** (86.67%) | / | 1. [Cat-] **Climb/Mount/Go/Walk/Jump** (75.33%) 2. [Cat-] (Can’t/doesn’t) **Come 1** (down) (38.67%) 3. [Girl-] **See/Find/Realise** (31.33%) 4. [Girl-] **Think/Want** (23.33%) 5. [Girl-] **Rescue/Capture/Catch/Hold/Take** (30.00%) 6. [Girl-] **Shout/Yell/Lure** (24.00%) 7. [Girl-] **Ask/Find/Inform/Request/ Ask (for help)** (29.33%) 8. [Cat-] **Come 2** (down) (36.00%) | 1. [Climb-] **Upon 1/Up**   OR **On 1** [the tree] (80.00%) |
| 1. The man stuck on the tree | 1. **Man 3** [-climb] (90.67%) 2. [Rescue-] **Cat 4** (58.00%) 3. **Dog** [-chase] (84.00%) 4. [-Chase] **Man 4** (41.33%) 5. **Man 5** [-can’t get down] (39.33%) | 1. **Tree 2** (76.67%) | 1. [Bring-] **Ladder 1** (42.00%) 2. **Ladder 2** [-fall] (30.67%) | 1. [Man-] **Bring/Take/Use** (40.67%) 2. [Man-] **Climb/Mount/Go** (up)**/Jump/Run** (86.00%) 3. [Man-] **Want/Think/Try/Wish** (42.00%) 4. [Man-] **Help** (21.33%) 5. [Man-] **Rescue/Catch/Hold/Get/Take /Drive** (50.00%) 6. [Cat-] **Come 3** (down) (27.33%) 7. [Ladder-] **Fall/Drop** (32.67%) 8. [Dog-] **Chase/Drive/Catch** (28.00%) 9. [Dog-] **Bark/Yell** (56.67%) 10. [Man-] **Scare/Frighten** (21.33%) 11. [Man-] (Can’t/doesn’t) **Come 4** (down) (56.00%) | 1. **However** [-dog chases man/ ladder falls] (38.67%) 2. [Climb-] **Upon 1/Up**   OR **On 1** [the tree] (75.33%)   1. Under/below [tree-] (28.00%) |
| 1. The firemen come | 1. **Firemen/Fire department** [-rescue] (92.67%) 2. [-Rescue] **Man 6/ Them** (84.67%) 3. [-Rescue] **Cat 5** (38.00%) | / | 1. [Bring-] **Ladder 3** (52.00%) | 1. [Man/Girl-] **Phone/Call** (30.00%) 2. **Ask/Call/Find/Bother/Alarm** [-Firemen] (62.00%) 3. [Firemen-] **Come/Go/Rush/Arrive** (79.33%) 4. [Firemen-] **Bring/Take** (50.00%) 5. [消防員-] **Rescue/Save/Help/Escort** (96.67%) 6. [男人-] **Come 5** (down) (52.67%) | 1. **Finally/Lastly/ Eventually** [-firemen come] (29.33%) |

Supplementary Table 8. Standard information content units (ICUs) for the sequential 4-picture description - “*Broken window*”.

| Scenario | ICUs (frequency) | | | | | | | | |  |
| --- | --- | --- | --- | --- | --- | --- | --- | --- | --- | --- |
|  | Subjects | Places | | | Objects | Actions | | | Others |  |
| Cantonese version | | | | | | | | | |  |
| 1. Introducing the boy who is playing football | 1. **小朋友1/男仔/細路仔/後生仔/小孩**[-踢波] (96.00%) | 1. **公園/花園** (22.00%) | | | / | 1. **踢**[-足球]/**踢波** (97.33%) | | | / |  |
| 1. The ball breaks the window | 1. **男人1/男子/屋主/主人/鄰居/爸爸/大人/住戶/大叔/陳生/人哋/人** (50.00%) 2. **小朋友2**[-踢] (33.33%) | 1. **屋/屋企/住宅/單位/屋苑/家/大廈** (75.33%) | | | 1. [打爛-]**窗1/窗戶/窗口/窗門** (80.67%) 2. **波1/足球**[-打爛窗] (52.00%) 3. [打爛-]**玻璃1**(45.33%) 4. **波2**[-跌入屋] (22.00%) | 1. **踢/射/抽**[-波] (78.00%) 2. **打爛/打破/撞爛/踢穿/踢爆/踢爛/穿/破壞** [-窗] (70.67%) 3. [波-]**飛/跌/入/去/彈/走** (50.00%) | | | 1. **唔小心/唔覺意/無意中/意外地/錯腳/唔好彩**[-踢] (43.33%) 2. [屋-]**入面/裡面** (39.33%) |  |
| 1. Condition inside the house | 1. **男人2**[-坐] (78.67%) | 1. **屋/屋企/家居** (32.00%) | | | 1. **波3** [-打爛] (36.67%) 2. [打爛-] **燈/檯燈/燈座/燈罩** (34.67%) 3. [打爛-] **窗2/玻璃2** (23.33%) | 1. **爛/打爛/踢爛/撞爛/撞跌/打斜** [-燈] (36.00%) 2. [男人-] **坐** (30.67%) 3. [男人-] **見/睇見/發現/發覺** (22.67%) | | | / |  |
| 1. The man’s reaction | 1. **男人3** [-執] (64.67%) | / | | | 1. [執-] **波4**(60.00%) 2. [望-] **窗3**(54.00%) 3. [踢-] **波5**(20.00%) | 1. **望/睇** [-窗] (74.00%) 2. [男人-] **執/攞/拎/揸/接** (63.33%) 3. **望/睇/搵** [-係邊個] (41.33%) 4. [邊個-] **踢/踢爛/踢波/打/射/做** (38.67%) 5. [男人-] **企/起** (21.33%) | | | 1. [望-] **外面/出面/出去/下面/落去/出** (53.33%) 2. **邊個** [-踢] (44.00%) 3. [踢-] **入嚟/上嚟** (22.00%) |  |
| English version | | | | | | | | | | |
| 1. Introducing the boy who is playing football | 1. **Child 1/Boy/Kid** [-playing] (96.00%) | | 1. **Playground/Yard**   (22.00%) | / | | | 1. **Kick**/**Play** [-football] (97.33%) | / | | |
| 1. The ball breaks the window | 1. **Man 1/Guy /Master/Adult/ Father/Uncle/ Neighbour/ Mr. Chan** [-house] (50.00%) 2. **Child 2** [-kicking] (33.33%) | | 1. **House/Home/Flat/Tenement/ Apartment/ Building** (75.33%) | 1. [Breaking-]**window/ casement** (80.67%) 2. **Ball 1** [-breaking] (52.00%) 3. [Breaking-]**glass** (45.33%) 4. **Ball 2**[-fall] (22.00%) | | | 1. **Kick/Shoot/Strike** [-ball] (78.00%) 2. **Break/Shatter/ Smash/Crack/ Splinter/Destroy** [-window] (70.67%) 3. [Ball-]**fall/fly/get/ bounce** (50.00%) | 1. **Carelessly/ Recklessly/ Accidentally/ Unfortunately** [-kick] (43.33%) 2. **Into** [-house] (39.33%) | | |
| 1. Condition inside the house | 1. **Man 2** [-sitting] (78.67%) | | 1. **House/Home** (32.00%) | 1. **Ball 3** [-breaking] (36.67%) 2. [Breaking-] **lamp/ light/lampshade** (34.67%) 3. [Breaking-] **window / glass** (23.33%) | | | 1. **Break/Smash/Hit/ Strike/Knock down** [-lamp] (36.00%) 2. [Man-] **sit** (30.67%) 3. [Man-] **see/find/ discover/figure** (22.67%) | / | | |
| 1. The man’s reaction | 1. **Man 3** [-picking up] (64.67%) | | / | 1. [Picking up-] **ball 4** (60.00%) 2. [Looking-] **window 3** (54.00%) 3. [Kicking-] **ball 5** (20.00%) | | | 1. **Look/Gaze** [-window] (74.00%) 2. [Man-] **grab**/**pick up/ take/catch** (63.33%) 3. **See/Search** [-who did] (41.33%) 4. [Who-] **kick/ shoot/break/hit/did** (38.67%) 5. [Man-] **stand up/get up** (21.33%) | 1. [Look-] **out/ outside/down** (53.33%) 2. **Who** [-kick] (44.00%) 3. [Kick-] **inside/ up** (22.00%) | | |

Supplementary Table 9. Standard information content units (ICUs) for the sequential 6-picture description - “*Refuse umbrella*”.

| Scenario | ICUs (frequency) | | | | |
| --- | --- | --- | --- | --- | --- |
|  | Subjects | Places | Objects | Actions | Others |
| Cantonese version | | | | | |
| 1. The mother asks the child to bring umbrella | 1. **小朋友1/男仔/阿仔/小明/女仔/妹妹** [-返學] (53.02%) 2. **媽媽1/媽咪/女人** [-叫/畀] (97.32%) 3. [叫/畀-] **小朋友2** (90.60%) | / | 1. [-帶] **遮1/雨遮/雨傘** (87.25%) 2. [-出] **門口1/門** (20.13%) | 1. [媽媽-] **叫/提/要/提醒/叮囑/建議/吩咐** (60.40%) 2. [媽媽-] **畀** (20.13%) 3. [媽媽-] **話1/講** (24.16%) 4. [小朋友-] **帶1/攞/拎/搦/揸/擔** (89.26%) 5. [小朋友-] **返學1/上學** (62.42%) 6. [小朋友-] **出街1/出去/出門** (30.20%) 7. **落雨1/落** (32.21%) | 1. **會/想/可能/應該** [-落雨] (22.15%) |
| 1. The child refuses | 1. **小朋友3** [-話(唔要)/(冇)帶] (87.25%) | / | 1. [-帶] **遮2** (30.20%) | 1. [小朋友-] **話2** (61.07%) 2. [小朋友-] **唔需要/要/使** (65.10%) 3. [小朋友-] **唔/冇帶2** (54.36%) |  |
| 1. There is a rain | 1. **小朋友4** [-出門/返學] (42.28%) | / | 1. [-落] **雨** (38.26%) 2. [-出] **門口2** (30.87%) | 1. [小朋友-] **出/出去/出門/離開/落** (61.07%) 2. [小朋友-] **行/行路/行行吓** (43.62%) 3. **落雨2** (96.64%) | 1. **點知/突然/居然** [-落雨] (48.99%) 2. **真係/果然** [-落雨] (46.98%) 3. [行-] **途中/一半/半路** (26.17%) 4. **大** [-雨] (40.94%) |
| 1. The child gets wet | 1. **小朋友5** [-走/返/揼濕] (59.73%) | 1. **屋企** (52.35%) | 1. **身1** [-濕] (25.50%) | 1. [小朋友-] **走/跑/衝/趕** (42.28%) 2. [小朋友-] **返** (40.27%) 3. [小朋友-] **揼/淋/揼濕/淋濕** (40.94%) 4. [小朋友-] **濕** (40.94%) |  |
| 1. The mother’s reaction | 1. **媽媽2** [-鬧/話/見/嬲/(唔)高興] (80.54%) 2. [鬧/話/見/叫-] **小朋友 6** (53.69%) | 1. **屋企** (34.23%) | 1. **身2** [-濕] (22.82%) 2. [-帶] **遮3** (30.87%) | 1. [媽媽-] **鬧/話/責備/責罰/怪責** (36.24%) 2. [媽媽-] **見/望/發覺** (27.52%) 3. [媽媽-] **嬲/唔高興/唔開心** (33.56%) 4. [小朋友-] **返** (44.97%) 5. [小朋友-] **濕** (40.94%) 6. [小朋友-] **帶3** (33.56%) |  |
| 1. Resolution | 1. **小朋友7** [-帶/聽/覺得/換/返學] (71.81%) 2. **媽媽3** [-講/畀/幫] (20.81%) | / | 1. [-帶] **遮4** (88.59%) | 1. [小朋友-] **帶4** (86.58%) 2. [小朋友-] **返學2** (48.32%) 3. [小朋友-] **出街2** (31.54%) | 1. **結果/最後/後尾/卒之/終於** (24.83%) |
| English version | | | | | |
| 1. The mother asks the child to bring umbrella | 1. **Child 1/ Boy/Son/Siu-Ming/Girl/Little sister** [-goes to school] (53.02%) 2. **Mother 1/Mom/ Woman** [-asks/gives] (97.32%) 3. [Ask/give-] **Child 2** (90.60%) | / | 1. [-Bring] **Umbrella 1** (87.25%) 2. [-go] **Doorway 1/Door** (20.13%) | 1. [Mother-] **Ask/remind/let/ urge/suggest/order** (60.40%) 2. [Mother-] **Give** (20.13%) 3. [Mother-] **Say/Tell** (24.16%) 4. [Child-] **Bring 1/Take/Get/Grab** (89.26%) 5. [Child-] **Back/Go to school 1** (62.42%) 6. [Child-] **Go out /Get on street 1** (30.20%) 7. **Rain 1** (32.21%) | 1. **Will/May** [-rain] (22.15%) |
| 1. The child refuses | 1. **Child 3** [-don’t want/didn’t bring] (87.25%) | / | 1. [-Bring] **Umbrella 2** (30.20%) | 1. [Child-] **Say/Tell** (61.07%) 2. [Child-] **Don’t need/want** (65.10%) 3. [Child-] **Didn’t bring 2** (54.36%) |  |
| 1. There is a rain | 1. **Child 4** [-go out/back to school] (42.28%) | / | 1. **Rain** (38.26%) 2. [-go] **Doorway 2** (30.87%) | 1. [Child-] **Go/get out/leave/get down/get on** (61.07%) 2. [Child-] **Walk** (43.62%) 3. **Rain 2** (96.64%) | 1. **Unexpectedly/ Suddenly/Surprisingly** [-rain] (48.99%) 2. **Really/Actually** [-rain] (46.98%) 3. [Walk-] **On the way/ halfway/en route** (26.17%) 4. **Heavy** [-rain] (40.94%) |
| 1. The child gets wet | 1. **Child 5** [-run/back home/get wet] (59.73%) | 1. **Home** (52.35%) | 1. **Body 1** [-get wet] (25.50%) | 1. [Child-] **Run/Sprint/Rush/Dash** (42.28%) 2. [Child-] **Back** (40.27%) 3. [Child-] **Get wet/Drenched/Soaked** (40.94%) 4. [Child-] **Wet** (40.94%) |  |
| 1. The mother’s reaction | 1. **Mother 2** [-blame/ask/saw/ angry/unhappy] (80.54%) 2. [Blame/ask/saw] **Child 6** (53.69%) | 1. **Home** (34.23%) | 1. **Body 2** [-get wet] (22.82%) 2. [-Bring] **Umbrella 3** (30.87%) | 1. [Mother-] **Blame/Charge/Punish/ Criticize** (36.24%) 2. [Mother-] **See/Look/Find** (27.52%) 3. [Mother-] **Angry/Unhappy** (33.56%) 4. [Child-] **Back** (44.97%) 5. [Child-] **Wet** (40.94%) 6. [Child-] **Bring 3** (33.56%) |  |
| 1. Resolution | 1. **Child 7** [-Bring/Listen/Think/ Change/Back] (71.81%) 2. **Mother 3** [-Say/Give/Help] (20.81%) | / | 1. [-Bring] **Umbrella 4** (88.59%) | 1. [Child-] **Bring** **4** (86.58%) 2. [Child-] **Go/Back to school** **2** (48.32%) 3. [Child-] **Go out /Get on street 2** (31.54%) | 1. **Finally/At last/Lastly/ In the end** (24.83%) |

Supplementary Table 10. Standard information content units (ICUs) for the procedural description - “*Egg ham sandwich*”.

| Scenario | ICUs (frequency) | | |
| --- | --- | --- | --- |
|  | Objects | Actions | Others |
| Cantonese version | | | |
| 1. Prologue | 1. [整-] **雞蛋1** (22.67%) 2. [整-] **火腿1** (23.33%) 3. [整-] **三文治/腿蛋治1** (33.33%) 4. [預備-]**雞蛋2** (20.67%) 5. [預備-]**火腿2** (20.67%) 6. [預備-]**麵包/方包1** (22.67%) | 1. **整/做** [-三文治] (29.33%) 2. **預備/攞/用/買/有** [-雞蛋/火腿/麵包] (22.67%) | / |
| 1. Handling the bread | 1. [烘-] **麵包2** (30.67%) | 1. **烘/焗** [-麵包] (22.33%) | / |
| 1. Handling the egg | 1. [打-] **雞蛋3** (52.67%) 2. [攪勻-] **雞蛋4** (37.33%) 3. [煎-] **雞蛋5** (83.33%) 4. [放-] **鑊/煎peng1/peng1/鍋**(42.00%) 5. [落-] **油**(24.67%) | 1. **打/打開/打爛** [-蛋] (51.33%) 2. **faak3/打勻/攪勻** [-蛋] (41.33%) 3. **煎/煎熟** [-蛋] (80.67%) 4. **用/搵/攞/開/燒** [-鑊] (23.33%) 5. **放/擺/倒/落** [-鑊] (22.00%) 6. **落/擺/加** [-油] (22.67%) 7. **擺/放/倒/鋪** [-麵包上] (24.00%) | 1. **首先/先** (48.00%) 2. **跟住/然後/之後/再1** (62.67%) |
| 1. Handling the ham | 1. [煎-] **火腿3** (53.33%) 2. [放-] **火腿4** (26.00%) | 1. **煎/煎熟/烘/熱/炒** [-火腿] (62.00%) 2. **放/夾/擺/鋪** [-麵包上] (24.67%) | - - - 1. **跟住/然後/之後/再2** (54.00%) |
| 1. Finishing | 1. [放-] **雞蛋6** (46.00%) 2. [放-] **火腿5** (49.33%) 3. [放-] **麵包3** (59.33%) 4. [冚/夾-] **麵包4** (33.33%) 5. [完成-] **雞蛋7** (27.33%) 6. [完成-] **火腿6** (31.33%) 7. [完成-] **三文治2** (61.33%) | 1. **擺/放/落/鋪/加1** [-麵包上] (55.33%) 2. **擺2** [-麵包上] (22.00%) 3. **冚/夾/包/摺** [-麵包] (56.67%) 4. **完成/整好/做成/搞掂/變成/成為** [-腿蛋治] (40.67%) | 1. **跟住/然後/之後/再3** (53.33%) 2. [放-] **上/上去/上面/面** (33.33%) |
| English version | | | |
| 1. Prologue | 1. [Make-] **Egg 1** (22.67%) 2. [Make-] **Ham 1** (23.33%) 3. [Make-] **Sandwich 1** (33.33%) 4. [Prepare-] **Egg** **2** (20.67%) 5. [Prepare-] **Ham** **2** (20.67%) 6. [Prepare-] **Bread/Toast 1** (22.67%) | 1. **Make/Do** [-sandwich] (29.33%) 2. **Prepare/Take/Use/Buy/Have** [-egg/ham/bread] (22.67%) | / |
| 1. Handling the bread | 1. [Toast-] **Bread 2** (30.67%) | 1. **Toast/Bake** [-bread] (22.33%) | / |
| 1. Handling the egg | 1. [Crack-] **Egg** **3** (52.67%) 2. [Whip-] **Egg** **4** (37.33%) 3. [Fry-] **Egg 5** (83.33%) 4. [Put-] **Frypan/Pan/ Skillet**(42.00%) 5. [Put-] **Oil** (24.67%) | 1. **Break/Crack** [-egg] (51.33%) 2. **Whip/Beat/Whisk** [-egg] (41.33%) 3. **Pan-fry** [-egg] (80.67%) 4. **Use/Find/Take/Heat** [-pan] (23.33%) 5. **Put/Pour** [-pan] (22.00%) 6. **Put/Add** [-oil] (22.67%) 7. **Put/Lay/Place/Spread** [-on the bread] (24.00%) | 1. **First** (48.00%) 2. **Then/After that/Next 1** (62.67%) |
| 1. Handling the ham | 1. [Fry-] **Ham 3** (53.33%) 2. [Put-] **Ham 4** (26.00%) | 1. **Pan-fry**/**Fry**/**Heat** [-ham] (62.00%) 2. **Put/Lay/Place/Clip** [-on the bread] (24.67%) | 1. **Then/After that/Next 2** (54.00%) |
| 1. Finishing | 1. [Put-] **Egg** **6** (46.00%) 2. [Put-] **Ham** **5** (49.33%) 3. [Put-] **Bread 3** (59.33%) 4. [Cover/Fold-] **Bread 4** (33.33%) 5. [Finish-] **Egg 7** (27.33%) 6. [Finish-] **Ham 6** (31.33%) 7. [Finish-] **Sandwich 2** (61.33%) | 1. **Put/Place/Add/Lay 1** [-on the bread] (55.33%) 2. **Put 2** [-on the bread] (22.00%) 3. **Cover/Fold/Wrap** [-Bread] (56.67%) 4. **Finish/Complete/Done/Turn into/Become** [-sandwich] (40.67%) | 1. **Then/After that/Next 3** (53.33%) 2. [Put-] **On/Onto/Upon/On top of** (33.33%) |

Supplementary Table 11. Standard main concepts (MCs) for the real-photo single-picture description - “*Flood*”.

| **Real Photo Single Picture Description – Flood** | | |
| --- | --- | --- |
| MC | Version |  |
| 1 | Version 1 | A flood **occurred**.  **發生** 水災  ***faat3 saang1*** *seoi2 zoi1* |
|  | Version 2 | The girl was **kayaking/hiking**.  女仔 **划艇/行山/遠足**  *neoi5 zai2* ***waak6 teng5/hang4 saan1/jyun5 zuk1*** |
|  | Version 3 | The girl was **rafting/playing** rapids.  女仔 **玩** 激流  *neoi5 zai2* ***waan2*** *gik1 lau4* |
| 2 | Version 1 | The girl **fell** into the river.  女仔 **跌** 落 河  *neoi5 zai2* ***dit3*** *lok6 ho4* |
|  | Version 2 | The girl was **trapped/flushed** by the floodwater.  女仔 被 洪水 **圍困/沖走**  *neoi5 zai2 bei6 hung4 seoi2* ***wai4 kwan3/cung1 zau2*** |
|  | Version 3 | The girl **drowned**.  女仔 **遇溺**  *neoi5 zai2* ***jyu6 nik1*** |
| 3 | Version 1 | The man was **wearing** a life jacket.  男人 **著**緊 件 救生衣  *naam4 jan2* ***zyu3*** *gan2 gin6 gau3 saang1 ji1* |
|  | Version 2 | The man was **holding** onto a tree branch.  男人 **捉**住 枝 樹枝  *naam4 jan2* ***zuk1*** *zyu6 zi1 syu6 zi1* |
|  | Version 3 | The man was **climbing** down.  男人 **爬樹** 落去  *naam4 jan2* ***paa4 syu6*** *lok6 heoi3* |
|  | Version 4 | The man **swam** to there.  男人 **游水** 過去  *naam4 jan2* ***jau4 seoi2*** *gwo3 heoi3* |
|  | Version 5 | The man **jumped** into the water.  男人 **跳** 落 水  *naam4 jan2* ***tiu3*** *lok6 seoi2* |
| 4 | - | The man was **saving** the girl.  男人 喺度 **救** 個 女仔  *naam4 jan2 hai2 dou6* ***gau3*** *go3 neoi5 zai2* |

Note. The main verb for each main concept is **bolded**. All the essential information within a main concept is underlined.

Supplementary Table 12. Standard main concepts (MCs) for the line-drawing single-picture description - “*Cat rescue*”.

| **Line-Drawing Single Picture Description – Cat Rescue** | | |
| --- | --- | --- |
| MC | Version |  |
| 1 | Version 1 | The girl/man/cat was **playing** in the park.  女仔/男人/貓 喺 公園 **玩**  *neoi5 zai2/naam4 jan2/maau1 hai2 gung1 jyun2* ***waan2*** |
|  | Version 2 | The girl/man/cat **passed by/went to** the park.  女仔/男人/貓 **經過/去** 公園  *neoi5 zai2/naam4 jan2/maau1* ***ging1 gwo3/heoi3*** *gung1 jyun2* |
|  | Version 3 | The girl **rode** a bicycle.  女仔 **踩** 單車  *neoi5 zai2* ***caai2*** *daan1 ce1* |
|  | Version 4 | The girl **had** a cat.  女仔 **養**咗 隻 貓  *neoi5 zai2* ***joeng5*** *zo2 zek3 maau1* |
| 2 | - | The cat **climbed** onto the tree.  貓 **爬**咗 上 樹  *maau1* ***paa4*** *zo2 soeng5 syu6* |
| 3 | Version 1 | The girl was **rescuing/catching/shouting to** the cat.  女仔 喺度 **救/捉/叫** 隻 貓  *neoi5 zai2 hai2 dou6* ***gau3/zuk1/giu3*** *zek3 maau1* |
|  | Version 2 | The girl **cried** for help.  女仔 **求救**  *neoi5 zai2* ***kau4 gau3*** |
|  | Version 3 | The girl **shouted to** the man.  女仔 **叫** 個 男人  *neoi5 zai2* ***giu3*** *go3 naam4 jan2* |
| 4 | Version 1 | The man was **rescuing/catching** the cat.  男人 **救/捉** 隻 貓  *naam4 jan2* ***gau3/zuk1*** *zek3 maau1* |
|  | Version 2 | The man **brought/climbed** the ladder.  男人 **擔/爬** 梯  *naam4 jan2* ***daam1/paa4*** *tai1* |
| 5 | Version 1 | The man **climbed** onto the tree.  男人 **爬**咗 上 樹  *naam4 jan2* ***paa4*** *zo2 soeng5 syu6* |
|  | Version 2 | The man **sat** on the tree.  男人 **坐**咗 喺 樹 上面  *naam4 jan2* ***co5*** *zo2 hai2 syu6 soeng5 min6* |
| 6 | Version 1 | The dog was **chasing/barking at/biting** the man.  狗 **追/吠/咬** 個 男人  *gau2* ***zeoi1/fai6/ngaau5*** *go3 naam4 jan2* |
|  | Version 2 | The dog was **scratching** the tree.  狗 **搲** 棵 樹  *gau2* ***we2*** *po1 syu6* |
|  | Version 3 | The dog **knocked** **down** the ladder.  狗 **撞跌** 堂 梯  *gau2* ***zong6 dit3*** *tong4 tai1* |
|  | Version 4 | The dog **was** under the tree.  狗 **喺** 棵 樹 下面  *gau2* ***hai2*** *po1 syu6 haa6 min6* |
| 7 | Version 1 | The man could not **come down**.  男人 **落** 唔到 嚟  *naam4 jan2* ***lok6*** *m4 dou2 lai4* |
|  | Version 2 | The man was **stuck** on top of the tree.  男人 **困** 喺 樹 上面  *naam4 jan2* ***kwan3*** *hai2 syu6 soeng5 min6* |
|  | Version 3 | The man was **frightened/scared**.  男人 好 **驚**  *naam4 jan2 hou2* ***geng1*** |
| 8 | Version 1 | The girl/man/passerby **called** the police.  女仔/男人/途人 **報警**  *neoi5 zai2/naam4 jan2/tou4 jan4* ***bou3 ging2*** |
|  | Version 2 | The girl/man/passerby **looked for** the firemen.  女仔/男人/途人 **搵** 消防員  *neoi5 zai2/naam4 jan2/tou4* ***wan2*** *siu1 fong4 jyun4* |
| 9 | Version 1 | The firemen **came**.  消防員 **嚟到**  *siu1 fong4 jyun4* ***lai4 dou3*** |
|  | Version 2 | The firemen **brought** a ladder.  消防員 **擔** 梯  *siu1 fong4 jyun4* ***daam1*** *tai1* |
| 10 | - | The firemen **rescued** them/the man.  消防員 **救** 男人/佢咃  *siu1 fong4 jyun4* ***gau3*** *naam4 jan2/keoi5 dei6* |

Note. The main verb for each main concept is **bolded**. All the essential information within a main concept is underlined.

Supplementary Table 13. Standard main concepts (MCs) for the sequential 4-picture description - “*Broken window*”.

| **Sequential 4-Picture Description (Broken Window)** | | |
| --- | --- | --- |
| MC | Version |  |
| 1 | Version 1 | The child was **playing** football.  小朋友 **踢波**  *siu2 pang4 jau5* ***tek3 bo1*** |
|  | Version 2 | The child was **kicking** a ball.  小朋友 **踢** 個 波  *siu2 pang4 jau5* ***tek3*** *go3 bo1* |
| 2 | - | He **kicked** the ball carelessly/strongly/mistakenly.  佢 唔小心/大力/錯腳 一 **踢**  *keoi5 m4 siu2 sam1/daai6 lik6/co3 goek3 jat1* ***tek3*** |
| 3 | Version 1 | The ball **broke** the window/glass.  個 波 **打爛**咗 個 窗/玻璃  *go3 bo1* ***daa2 laan6*** *zo2 go3 coeng1/bo1 lei4* |
|  | Version 2 | (The child) **broke** the window/glass.  (小朋友) **踢爛**咗 個 窗/玻璃  *(siu2 pang4 jau5)* ***tek3 laan6*** *zo2 go3 coeng1/bo1 lei4* |
| 4 | Version 1 | The ball **fell** into the man’s house.  個 波 **飛**咗 入 個 男人 屋企  *go3 bo1* ***fei1*** *zo2 jap6 go3 naam4 jan2 nguk1 kei5* |
|  | Version 2 | The ball **broke** the lamp inside the house.  個 波 **打爛**埋 屋 入面 個 檯燈  *go3 bo1* ***daa2 laan6*** *maai4 nguk1 jap6 min6 go3 toi4 dang1* |
| 5 | Version 1 | The man **saw** the ball.  男人 **見**到 個 波  *naam4 jan2* ***gin3*** *dou3 go3 bo1* |
|  | Version 2 | The man was **sitting** on a sofa.  男人 **坐**咗 喺 梳化 度  *naam4 jan2* ***co5*** *zo2 hai2 so1 faa3 dou6* |
|  | Version 3 | The man was **watching** TV.  男人 **睇**緊 電視  *naam4 jan2* ***tai2*** *gan2 din6 si6* |
|  | Version 4 | The man was **shocked**.  男人 好 **驚訝**  *naam4 jan2 hou2* ***ging1 ngaa5*** |
|  | Version 5 | The man was **angry**.  男人 好 **嬲**  *naam4 jan2 hou2* ***nau1*** |
| 6 | - | He **picked** up/**caught** the ball.  佢 **執** 起 / **接**住 個 波  *keoi5* ***zap1*** *hei2 /* ***zip3*** *zyu6 go3 bo1* |
| 7 | Version 1 | (He) **looked** out of the window.  (佢) **望** 出 窗 外  *(keoi5)* ***mong6*** *ceot1 coeng1 ngoi6* |
|  | Version 2 | (He) **looked** at the window.  (佢) **望**住 個 窗  *(keoi5)* ***mong6*** *zyu6 go3 coeng1* |
|  | Version 3 | (He) **walked** towards the window.  (佢) **行** 去 窗 度  *(keoi5)* ***hang4*** *heoi3 coeng1 dou6* |
| 8 | - | (He) **searched** for/**blamed** the child.  (佢) **搵/鬧** 個 小朋友  *(keoi5)* ***wan2/naau6*** *go3 siu2 pang4 jau5* |

Note. The main verb for each main concept is **bolded**. All the essential information within a main concept is underlined.

Supplementary Table 14. Standard main concepts (MCs) for the sequential 6-picture description - “*Refuse umbrella*”.

| **Sequential 6-Picture Description (Refuse Umbrella)** | | |
| --- | --- | --- |
| MC | Version |  |
| 1 | - | The child **went** out/**went** to school.  小朋友 **出街/返學**  *siu2 pang4 jau5* ***ceot1 gaai1/faan1 hok6*** |
| 2 | - | Mother **reminded** the child.  媽媽 **提醒** 小朋友  *maa4 maa1* ***tai4 sing2*** *siu2 pang4 jau5* |
| 3 | Version 1 | (Mother reminded the child to) **bring** umbrella.  (媽媽 提醒 小朋友) **帶** 遮  *(maa4 maa1 tai4 sing2 siu2 pang4 jau5)* ***daai3*** *ze1* |
|  | Version 2 | Mother **gave** the child an umbrella.  媽媽 **畀** 遮 小朋友  *maa4 maa1* ***bei2*** *ze1 siu2 pang4 jau5* |
| 4 | Version 1 | The child did not **bring** (the umbrella).  小朋友 唔 **帶** (遮)  *siu2 pang4 jau5 m4* ***daai3*** *(ze1)* |
|  | Version 2 | The child **refused** (the mother).  小朋友 **拒絕** (媽媽)  *siu2 pang4 jau5* ***keoi5 zyut6*** *(maa4 maa1)* |
| 5 | Version 1 | The child **went** outside/**went** to school.  小朋友 **出門/返學**  *siu2 pang4 jau5* ***ceot1 mun4/faan1 hok6*** |
|  | Version 2 | The child was **walking**.  小朋友 **行行吓**  *siu2 pang4 jau5* ***hang4 hang4 haa5*** |
| 6 | - | It suddenly **rained**.  突然 **落雨**  *dat6 jin4* ***lok6 jyu5*** |
| 7 | Version 1 | The child **ran**.  小朋友 **跑**  *siu2 pang4 jau5* ***paau2*** |
|  | Version 2 | The child **went** back home.  小朋友 **返** 屋企  *siu2 pang4 jau5* ***faan1*** *nguk1 kei5* |
| 8 | Version 1 | The child was **wet**.  小朋友 **濕**晒  *siu2 pang4 jau5* ***sap1*** *saai3* |
|  | Version 2 | The child got **soaked** in the rain.  小朋友 畀 雨 **淋**  *siu2 pang4 jau5 bei2 jyu5* ***lam4*** |
| 9 | Version 1 | Mother **blamed** the child.  媽媽 **鬧** 小朋友  *maa4 maa1* ***naau6*** *siu2 pang4 jau5* |
|  | Version 2 | Mother was **angry**.  媽媽 好 **嬲**  *maa4 maa1 hou2* ***nau1*** |
| 10 | Version 1 | The child **brought** an umbrella.  小朋友 **帶** 遮  *siu2 pang4 jau5* ***daai3*** *ze1* |
|  | Version 2 | Mother **gave** the child an umbrella.  媽媽 **畀** 遮 小朋友  *maa4 maa1* ***bei2*** *ze1 siu2 pang4 jau5* |
| 11 | - | The child **went** out/**went** to school (again).  小朋友 (再) **出街/返學**  *siu2 pang4 jau5 zoi3* ***ceot1 gaai1/faan1 hok6*** |

Note. The main verb for each main concept is **bolded**. All the essential information within a main concept is underlined.

Supplementary Table 15. Standard main concepts (MCs) for the procedural description - “*Egg ham sandwich*”.

| **Procedural Discourse – Egg Ham Sandwich** | | |
| --- | --- | --- |
| MC | Version |  |
| 1 | Version 1 | **Crack** the egg.  **打**  雞蛋  ***daa2*** *gai1 daan2* |
|  | Version 2 | **Whip** the egg.  **攪勻**  雞蛋  ***gaau2 wan4*** *gai1 daan2* |
| 2 | - | **Pan-fry/fry/cook** the egg.  **煎/炒/煮**  雞蛋  ***zin1/caau2/zyu2*** *gai1 daan2* |
| 3 | - | **Pan-fry/fry/cook/heat** the ham.  **煎/炒/煮/烘**  火腿  ***zin1/caau2/zyu2/hong3*** *gai1 daan2* |
| 4 | Version 1 | **Put** ham and egg on/above the bread.  **放** 火腿 同 雞蛋 喺 麵包 上面  ***fong3*** *fo2 teoi2 tung4 gai1 daan2 hai2 min6 baau1 soeng5 min6* |
|  | Version 2 | **Put** the ingredients on/above the bread.  **放** 啲 材料 喺 麵包 上面  ***fong3*** *di1 coi4 liu2 hai2 min6 baau1 soeng5 min6* |
|  | Version 3 | **Insert/put** the ham and egg between the bread.  麵包 **夾** 火腿 同 雞蛋  *min6 baau1* ***gaap3*** *fo2 teoi2 tung4 gai1 daan2* |
|  | Version 4 | **Insert/put** the ingredients between the bread.  麵包 **夾** 材料  *min6 baau1* ***gaap3*** *coi4 liu2* |

Note. The main verb for each main concept is **bolded**. All the essential information within a main concept is underlined.
